# Mutual annotation‐based prediction of protein domain functions with Domain2GO

**DOI:** 10.1002/pro.4988

**Published:** 2024-05-16

**Authors:** Erva Ulusoy, Tunca Doğan

**Affiliations:** ^1^ Biological Data Science Lab, Department of Computer Engineering Hacettepe University Ankara Turkey; ^2^ Department of Bioinformatics Graduate School of Health Sciences, Hacettepe University Ankara Turkey

**Keywords:** biomolecular function prediction, CAFA challenge, expectation maximization, gene ontology, protein domains

## Abstract

Identifying unknown functional properties of proteins is essential for understanding their roles in both health and disease states. The domain composition of a protein can reveal critical information in this context, as domains are structural and functional units that dictate how the protein should act at the molecular level. The expensive and time‐consuming nature of wet‐lab experimental approaches prompted researchers to develop computational strategies for predicting the functions of proteins. In this study, we proposed a new method called Domain2GO that infers associations between protein domains and function‐defining gene ontology (GO) terms, thus redefining the problem as domain function prediction. Domain2GO uses documented protein‐level GO annotations together with proteins' domain annotations. Co‐annotation patterns of domains and GO terms in the same proteins are examined using statistical resampling to obtain reliable associations. As a use‐case study, we evaluated the biological relevance of examples selected from the Domain2GO‐generated domain‐GO term mappings via literature review. Then, we applied Domain2GO to predict unknown protein functions by propagating domain‐associated GO terms to proteins annotated with these domains. For function prediction performance evaluation and comparison against other methods, we employed Critical Assessment of Function Annotation 3 (CAFA3) challenge datasets. The results demonstrated the high potential of Domain2GO, particularly for predicting molecular function and biological process terms, along with advantages such as producing interpretable results and having an exceptionally low computational cost. The approach presented here can be extended to other ontologies and biological entities to investigate unknown relationships in complex and large‐scale biological data. The source code, datasets, results, and user instructions for Domain2GO are available at https://github.com/HUBioDataLab/Domain2GO. Additionally, we offer a user‐friendly online tool at https://huggingface.co/spaces/HUBioDataLab/Domain2GO, which simplifies the prediction of functions of previously unannotated proteins solely using amino acid sequences.

## 1 INTRODUCTION

To comprehend biological mechanisms and develop effective treatment strategies for diseases, it is necessary to have knowledge of both the molecular functions of proteins and their roles in large‐scale biological processes. Experimental function identification techniques cannot keep up with the increasing amount of sequence data due to their expensive and time‐consuming nature. The ever‐expanding output of high throughput sequencing is stored in public databases such as the UniProt Knowledgebase (UniProtKB) (UniProt Consortium, 2023), where the vast majority of protein sequences (%99, as of September 2022) are missing experimental or manually curated annotation.

Considering that the biological functions of proteins are multifaceted and there is a vast amount of scientific knowledge on this topic, it is important to define this knowledge in a systematic and machine‐readable way for standardization. There are various classification systems that have been created to meet this need. Gene ontology (GO) is the most extensive and widely used resource, providing a standardized vocabulary of information on the functions of gene products (Ashburner et al., 2000; The Gene Ontology Consortium et al., 2023). The GO defines different aspects of protein function in three sub‐ontologies (i.e., molecular function [MFO], biological process [BPO], and cellular component [CCO]), which contain a total of 43,791 terms (as of November 2021). GO terms of each sub‐ontology are hierarchically linked to each other with well‐defined relationships, forming a directed acyclic graph (DAG).

Protein family and domain databases such as InterPro (Hunter et al., 2009) stand as comprehensive resources of protein signatures, which can be used to infer the structure and function of unknown proteins. InterPro2GO mappings (Mitchell et al., 2015), created by the InterPro team, aim to provide high‐quality curated functional annotations directly for domains. Even though this approach has been used since 2002, the annotation coverage of InterPro v83.0 only remains at 41% (15,839 out of 38,345 InterPro entries are associated with at least one GO term).

A variety of computational approaches have been developed for predicting protein functions over the years (Doğan, 2018; Kulmanov & Hoehndorf, 2020; Rifaioglu et al., 2018, 2019; Unsal et al., 2022; Zhao et al., 2022), to serve the ultimate aim of attaining a complete understanding of biological mechanisms. The idea behind these approaches is that annotations can be transferred among proteins sharing similar characteristics (e.g., sequence, structure, protein‐protein interactions, expression profiles, etc.). One efficient class of methods utilizes the domain‐based approach to address the challenging task of protein function prediction. The domain composition of a protein is one of the key indicators of its function(s). There are earlier studies that aim to investigate similar domain compositions in different proteins in order to infer their functional properties. Forslund and Sonnhammer (2008) implemented this idea as a simple and intuitive tool that makes use of available domain architecture data to transfer annotations between distant proteins. While most of these methods consider only the domain content of proteins (Das et al., 2015; Rojano et al., 2022), there are studies that involve attributes such as order, position, and recurrence of domains to measure similarity between domain architectures (Doğan et al., 2016). Integrating multiple sequence‐related properties, including the domain architecture, using machine learning algorithms has been shown to improve the function prediction performance (Kulmanov & Hoehndorf, 2020; You et al., 2018).

The critical assessment of function annotation (CAFA) (Jiang et al., 2016; Radivojac et al., 2013; Zhou et al., 2019) is a time‐based challenge designed to meet the need for assessing the performance of protein function prediction approaches in a comprehensive and objective manner. At the beginning of each round of the CAFA challenge, organizers provide a target set that contains proteins whose functions are unknown. Participants predict the functions of proteins in this dataset and submit them. During a data collection period, target proteins that gain experimental annotation accumulate a benchmark set, which is then used to evaluate the performance of participating methods. The CAFA challenge has been widely adopted in the protein science community and has become a community‐driven worldwide benchmark effort with three completed and one ongoing installment as of September 2022.

Although each of these function prediction methods helps to bridge the gap between sequencing and function annotation capabilities, protein function prediction remains an open problem, as evidenced by recent CAFA experiments. A limitation of the machine learning‐based models, which are among the best performers in CAFA challenges, is that it is hard to comprehend the underlying decision mechanisms behind predictions. Therefore, most of these models are problematic in terms of interpretability. Another limitation is related to computational complexity. Although hybrid models that combine multiple data types and/or algorithms could achieve high prediction performance, they come at a high computational cost, which makes them incapable of scaling up to large‐scale datasets such as the entire UniProtKB.

**FIGURE 1:**
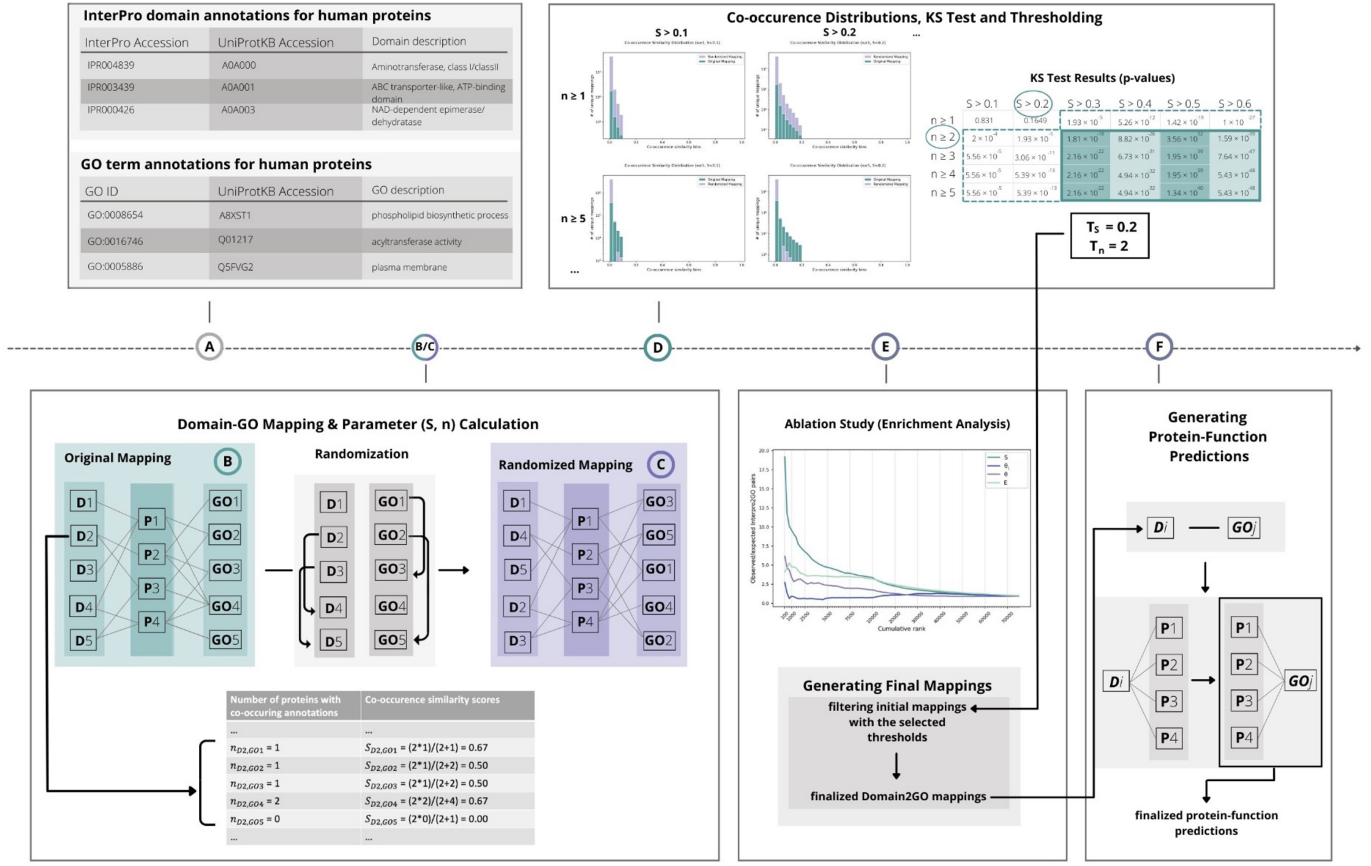
Schematic representation of the proposed method. (a) The source datasets were downloaded and organized; (b) initial mappings between the InterPro domains and GO terms were obtained, and the mapping parameters were calculated; (c) generation of the randomized annotation and mapping sets were constructed; (d) co‐occurrence similarity distributions were plotted, and thresholds were selected based on statistical resampling; (e) an ablation study was conducted by calculating the enrichment of top predictions ranked by different statistical measures and finalized Domain2GO mappings were generated by filtering initial mappings; (f) protein function predictions were generated by propagating Domain2GO mappings to target proteins.

To address the issues outlined above, we present a new method, Domain2GO, that utilizes the associations between protein domains and functions in order to infer GO term annotations of uncharacterized proteins (Figure 1). Domain2GO links two types of relational information to each other: domain‐protein and protein‐function, in order to obtain GO term predictions for both InterPro domains and full proteins. InterPro domains and GO terms that display statistically significant co‐occurrence patterns on different proteins are found to be related and included in our association list called “Domain2GO mappings.” Accordingly, these GO terms are used to annotate the proteins that contain the corresponding domains, resulting in protein function predictions. We evaluated the results of our method by comparing the obtained predictions with the expert curated domain‐function mappings from InterPro2GO (Mitchell et al., 2015). Additionally, we employed Domain2GO to predict the functions of the target proteins from the third version of the CAFA challenge (CAFA3) and compared the prediction performance against leading methods in this challenge, domain‐based approaches and baseline protein function prediction methods. This analysis aimed to assess Domain2GO's ability to generate protein annotations at a large scale as a secondary downstream task. As a use‐case study, we examined selected Domain2GO pairs to observe whether they possess biological relevance. The finalized Domain2GO mappings were used to obtain function predictions for the proteins in the whole UniProtKB/Swiss‐Prot database.

## 2 RESULTS AND DISCUSSION

### 2.1 Statistical analysis of the mappings

The original and randomized Domain2GO mapping generation process is explained in detail in Section 4.2. The procedure resulted in 2,069,796 unique initial/raw mappings for the original and 1,927,988 unique mappings for the randomized set, between 8642 domains and 28,420 GO terms. The numbers of Domain2GO pairs, domains, and GO terms in the original and randomized mapping sets at different co‐occurrence similarity thresholds are shown in Table S1.

**FIGURE 2:**
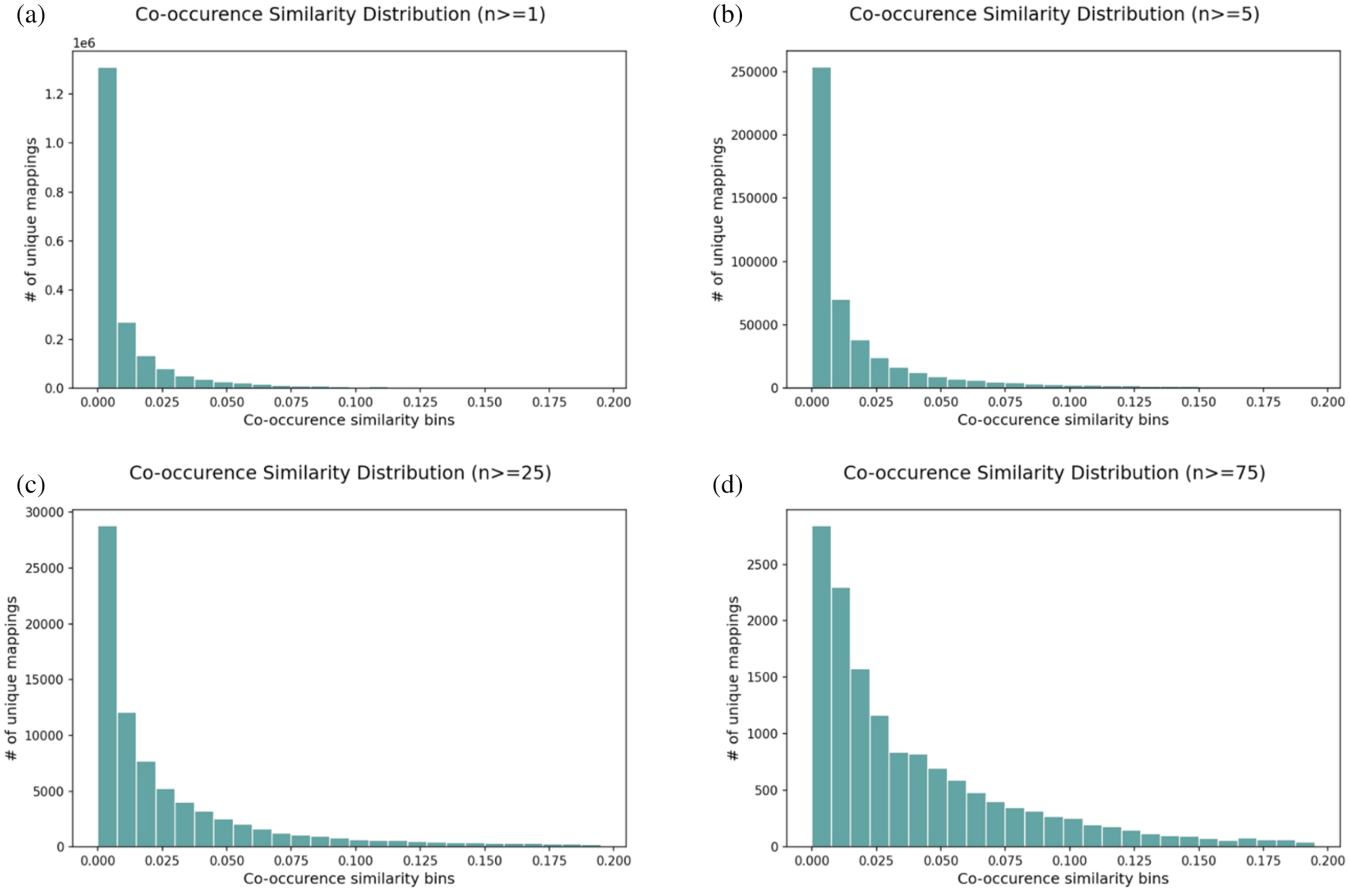
The co‐occurrence similarity distributions of the initial/raw original Domain2GO mappings. Each plot is drawn for a different threshold of the number of co‐annotated proteins (*n*); (a) *n* ≥ 1, (b) *n* ≥ 5, (c) *n* ≥ 25 (d) *n* ≥ 75.

Co‐occurrence similarity distributions for the original mapping set are shown in Figure 2 as histograms for selected *n* values. It has been observed that mappings with low co‐occurrence similarity, which have a high probability of being paired by chance, are accumulated in the distributions of low *n* values (e.g., 1 and 5). When higher *n* thresholds were applied, the number of these low‐confidence mappings was drastically reduced, indicating that the reliability of the mappings in the dataset has increased. 99.98% and 99.99% of the mappings with a co‐occurrence score lower than 0.0225 (i.e., the first three bins of the histograms) were eliminated at *n* ≥ 25 and *n* ≥ 75, respectively (Figure 2c,d). Regardless of this improvement, it is important to note that when extreme *n* thresholds (i.e., 25 and 75) were applied, only a small portion of the initial mapping set remained. Moreover, it is possible to observe mappings with extremely low *S* values even at these stringent *n* threshold values. These observations demonstrate the necessity of combining *S* and *n* values to filter out unreliable mappings.

We utilized the KS test to determine the optimal *S* and *n* threshold pair for distinguishing relevant mappings from the remainder. By combining arbitrarily selected *n* and *S* values, 30 co‐occurrence distributions were generated for both the original and randomized mapping sets (Table S2). The KS test was applied to the remaining data points from both distributions following the filtering operation by applying the corresponding *S* and *n* threshold pair. The minimum value pair that rejects the null hypothesis, which states that the two samples (i.e., the original and the randomized mapping set) are drawn from the same distribution, was selected to be used as finalized thresholds. As can be seen in Table S2, the minimum value pair that yielded the required statistical significance (i.e., *p*‐value <0.01) is *n* ≥ 2 and *S* > 0.1. The co‐occurrence distribution of the original mapping set containing pairs with n and S values lower than these thresholds shows no significant difference between the original and random mappings, and therefore, is considered unreliable. While this value pair seems appropriate for use as official thresholds, we selected *n* ≥ 2 and *S* > 0.2 to make a safe choice and eliminate all the initial Domain2GO mappings other than those with high reliability. Following this analysis, the finalized Domain2GO mapping set contained 26,696 associations between 4742 InterPro domains and 11,742 GO terms, which are available at https://github.com/HUBioDataLab/Domain2GO.

### 2.2 Ablation study (enrichment analysis)

In order to assess the prediction capabilities of different versions of the domain‐function mapping procedure, we employed three different measures calculated by the EM algorithm (as explained in Section 4.3) and compared their results to those of the co‐occurrence similarity‐based mapping. The first measure, which we abbreviate as “*θ*_*i*_,” is an initial estimate of the probability of association between a domain and a GO term (Equation 5). The EM algorithm was performed to refine this initial estimate for each domain‐GO term pair, and the output score is denoted as *θ*, and used as the second measure. The refined *θ* value is considered to be obtained at the point where the likelihood (Equation 6) stops increasing noticeably. Finally, we performed a likelihood ratio test by rerunning EM for each pair, this time excluding the given pair from the pool of competing domain‐GO term pairs (as explained in Section 4.3.2). The resulting likelihood ratio, which is denoted as *E*, stands as an evidence score for the given domain‐GO term association and is used as our third measure. To compare the prediction performance of each different statistical measure (i.e., *S*, *θ*_*i*_, *θ*, and *E*), ideally, the likelihood ratio test should have been performed for each Domain2GO pair in the initial set (2,069,796 mappings) by rerunning the EM until the likelihood values (Equation 6) stop increasing noticeably. However, it was only possible to perform this step for a subset of the initial dataset composed of 78,477 mappings between 21,466 GO terms and 6870 domains (obtained by filtering the initial set using a threshold of *S* ≥ 0.1) for 10 iterations due to high computational complexity (performing the same process for the full initial set would take 2272 days on a single CPU of a regular consumer‐level computer). Therefore, all the other measures were re‐calculated based on this subset for comparison. We iterated the EM algorithm 150 times to obtain the *θ* value, increasing the log‐likelihood from −2.2 × $10^{6}$ to the stopping point of −5.89 × $10^{5}$. For the likelihood ratio test, we re‐iterated the EM algorithm 10 times for each domain‐GO term pair, increasing the log‐likelihood from −2.2 × $10^{6}$ to the stopping point of −6 × $10^{5}$ in each re‐iteration.

**FIGURE 3:**
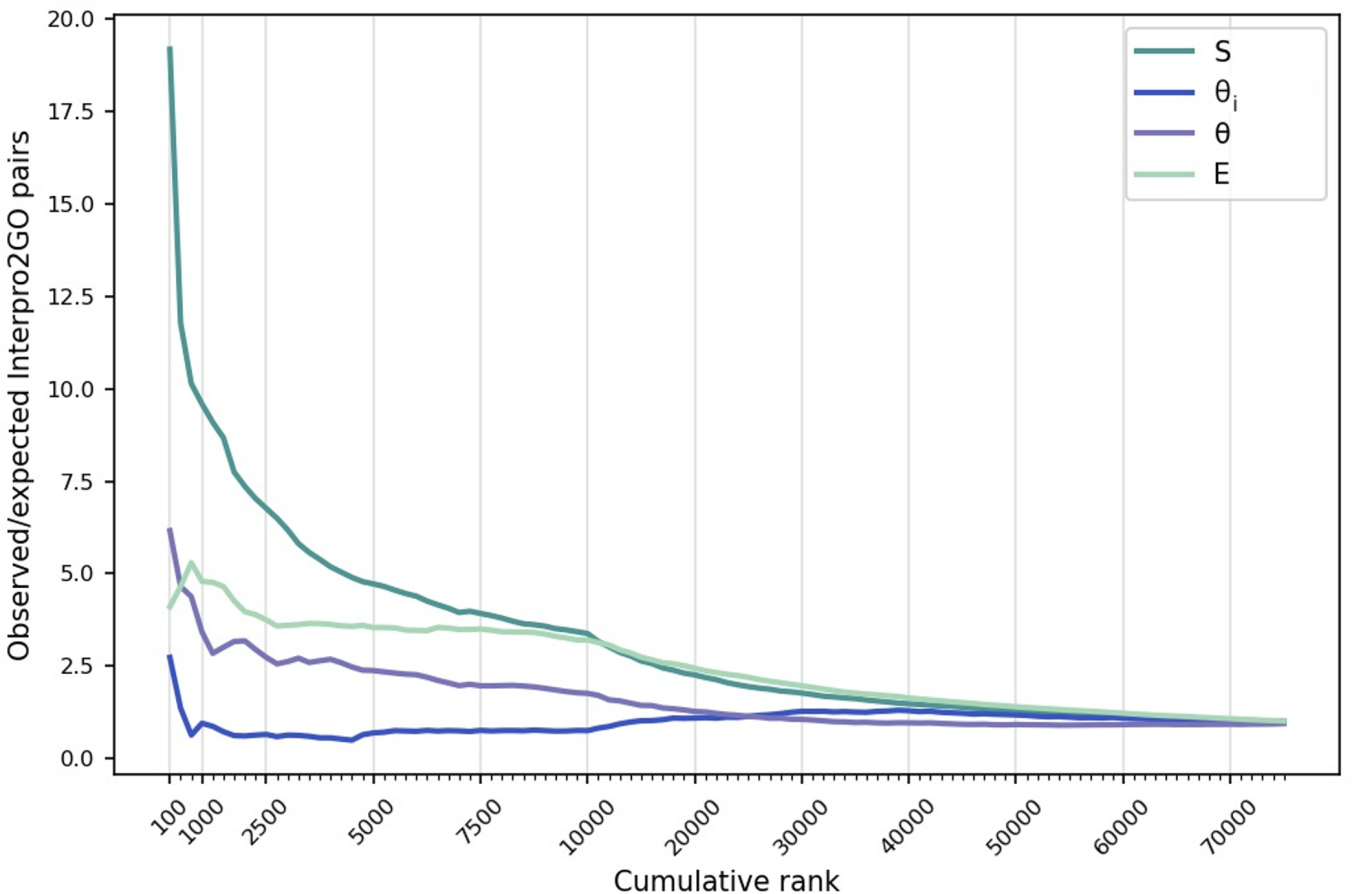
The enrichment of manually curated domain‐GO term associations in the top‐ranking Domain2GO mappings. Mappings are ranked by four different measures: co‐occurrence similarity (*S*), initial estimate of the association probability (*θ_i_*), final estimate of the association probability (*θ*) and evidence measure for the association (*E*). The ratio of observed/expected manual associations in the top K mappings (y‐axis) ranked by each of the four measures is plotted against the cumulative rank (x‐axis). Higher enrichment values are indicators of better performance.

An enrichment analysis was performed to evaluate which measure yields the most accurate associations. For this, we listed ranked predictions for each of the four measures (i.e., *S*, *θ*_*i*_, *θ*, and *E*). The number of ground truth domain‐GO term pairs (provided by manually curated InterPro2GO associations) in the top K predictions was calculated for each measure. The number of manually associated pairs that are anticipated to appear in the top K predictions (i.e., expected mappings) was then estimated using the ratio of pairs that are verified by InterPro2GO across the entire prediction list. Enrichment plots (i.e., the ratio of observed to expected mappings in top K domain‐GO term pairs) by each of the four measures (*S*, *θ*_*i*_, *θ*, and *E*) are displayed in Figure 3. Pairs ranked by the *θ*_*i*_ scores showed no significant enrichment at any rank cutoff. The enrichment was 6.1‐fold in the top 100 predictions when the pairs were ranked by *θ*, verifying the improved performance after EM optimization. Ranking by *E* increased the enrichment slightly further (from 3.4‐ to 4.7‐fold) in the top 1000. The ranking by *S* yielded the highest enrichment, with 19‐fold, in the top 100 predictions. The results suggest that the *S* score was able to identify significant domain‐GO term pairs better than *θ*_*i*_, *θ*, and *E*, especially at low‐rank cutoffs (in the top 100 to 10,000 predictions). However, if the likelihood ratio test could be performed on the full initial set with a sufficient number of iterations, it might be possible for the *E* score to compete with the *S* score. Unfortunately, we were unable to test this hypothesis due to the significant computational time required for the calculation of the *E* scores. Based on the results of the ablation study, we decided to continue our experiments with the mappings obtained from co‐occurrence similarity‐based (*S*) filtering.

### 2.3 Comparison with the manually curated associations

The InterPro team has generated a set of curated domain function annotations called “InterPro2GO,” by first matching the InterPro entries with UniProtKB proteins using sequence information and then assigning GO terms to them based on common features shared between the matched proteins (Mitchell et al., 2015). InterPro2GO mappings are manually curated, thus considered reliable. However, their coverage is low due to the time and labor‐intensive nature of manual curation. In its current form, there are 5881 manual associations between 3056 domains and 1524 GO terms in InterPro2GO (version: 2020/12).

To evaluate our method in terms of generating biologically relevant domain‐GO term associations, we compared the finalized Domain2GO mappings with InterPro2GO as a reference domain annotation system. For this, we calculated the correspondence between domain‐GO term pairs, which revealed that our predictions retrieved 737 out of 5881 InterPro2GO associations. When the correspondence between domains was evaluated, we observed that 60% of the domains in the manual associations were paired with at least one GO term in our predictions as well. We found that 30% of the domains in our predictions were also included in the InterPro2GO associations. Similar calculations were performed on GO terms, yielding a 67% and 9% correspondence rate, respectively. According to these results, Domain2GO managed to find connections to more than half of the domains and GO terms presented in InterPro2GO and many more.

Burge et al. (2012) followed a curation protocol in which they assigned GO terms to domains based solely on published experimental evidence of the domain's specific function, thereby omitting numerous promising associations. As a result, the specificity of the protocol increased while its coverage decreased. Many of the associations retrieved by Domain2GO contain GO terms that are closely related (on the directed acyclic graph of Gene Ontology) to GO terms annotated to the same domain in InterPro2GO. For example, Interpro2GO mappings contain an association between “histone acetylation” (GO:0016573) and “nuclear receptor coactivator, CREB‐bp‐like, interlocking” (IPR014744), which was one of the most frequent domains in the manual associations. One of the GO terms we predicted to be associated with IPR014744 was “histone H2B acetylation” (GO:0043969), which is directly related (with “is a” relation) to the manually associated GO:0016573 as its child term. This comparison demonstrates that the proposed method was able to; (1) retrieve many of the mappings in InterPro2GO and (2) provide more specific domain‐function associations.

Table S3 displays 50 arbitrarily selected domain‐GO term pairs that are considered highly reliable based on their respective *S* and *n* values. As the table shows, pairs with a high *S* score are commonly present in manually curated InterPro2GO mappings. When the correspondence between the manual associations and the high‐confidence mappings (i.e., pairs with an *S* ≥ 0.9) was calculated, it was found that 123 out of 737 high‐confidence mappings are found in InterPro2GO.

### 2.4 Biological relevance of the selected mappings—A case study

**FIGURE 4:**
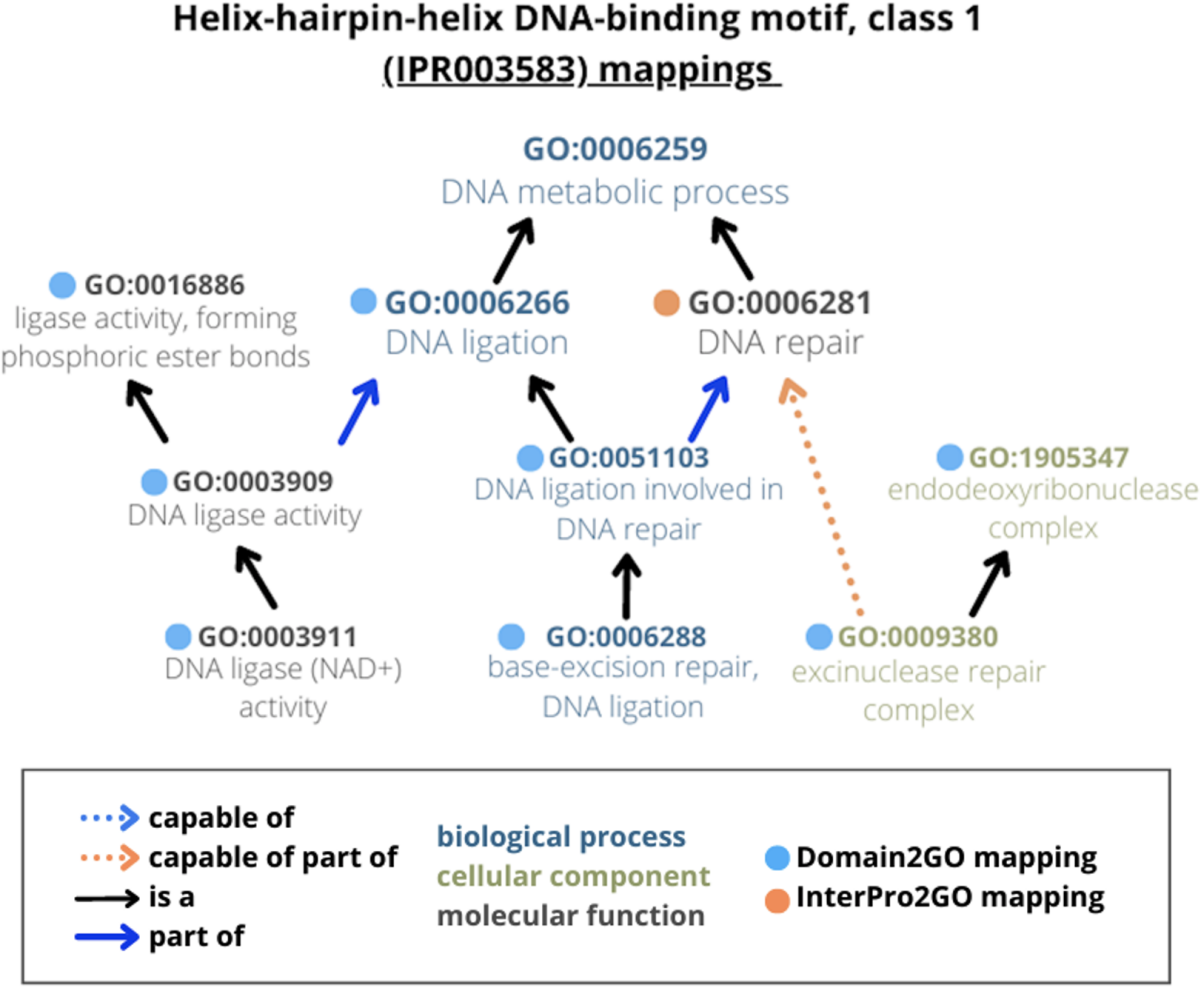
A case study comparing Domain2GO with curated InterPro2GO annotations on a selected domain. Case 1: GO terms associated with Helix‐hairpin‐helix DNA‐binding motif, class 1 (IPR003583) domain.

To evaluate the biological significance of our findings, we examined three Domain2GO mappings. The statistics of these domain‐GO term pairs are shown in the last three rows of Table S3 and the semantic relationships between predicted and manually annotated GO terms for the given domains are shown in Figure 4 for case 1, and in Figure S2 for cases 2 and 3.

The first case is a mapping between the domain “helix‐hairpin‐helix DNA‐binding motif, class 1” (IPR003583) and the GO biological process term “DNA ligation involved in DNA repair” (GO:0051103). These two terms were mapped to each other with *S* = 0.39 and *n* = 25. Helix‐hairpin‐Helix (HhH) is known as the most common nucleic‐acid binding module in repair proteins (Aravind et al., 1999). The role of HhH domain in repair processes is also verified by a manually curated InterPro2GO association between IPR003583 and the biological process term “DNA repair” (GO:0006281), which is related (i.e., with part_of relationship) to the mapped GO:0051103 term as its parent. GO:0051103 is defined as a process that “contributes to DNA repair, carried out by DNA ligase” in the UniProt‐GOA database (URL: https://www.ebi.ac.uk/QuickGO/term/GO:0051103). HhH was detected in DNA ligases at both sequence and structural levels by the elucidation of crystal structures (Doherty & Suh, 2000). Accordingly, the proteins that contain the domain IPR003583, and thus annotated to the function term GO:0051103 thanks to the corresponding Domain2GO mapping, are all DNA ligases from different organisms according to the information provided in UniProtKB (e.g., UniProt accessions: P15042, Q9WXV5, Q8A9C1, Q8DPS9, etc.). We also found a mapping between IPR003583 and the GO cellular component term “excinuclease repair complex” (GO:0009380), which is related (i.e., with capable_of_part_of relationship) to the manually associated GO:0006281 term, as its child. This is another clear example of Domain2GO associating domains with biologically relevant GO terms. Importantly, whereas InterPro2GO defines associations between the IPR003583 domain and two GO terms, one from the biological process and the other from the molecular function categories, the proposed method was able to define additional associations with cellular component terms (Figure 4). The biological relevance of the other two selected cases is discussed in the Supplementary Material document, Section 5 and Figure S2.

### 2.5 Protein function prediction performance evaluation

The main aim of Domain2GO is to predict the functions of domains, not full proteins. Nevertheless, it is interesting to examine the capabilities of Domain2GO in this regard. This is done using the competitive and established framework of the CAFA3 challenge (Zhou et al., 2019). We evaluated the complete protein function prediction performance of Domain2GO using the CAFA3 challenge benchmark dataset, which includes proteins that acquired manually curated GO associations during the annotation collection period. To ensure fairness and eliminate bias in our evaluation, we aligned our training data with the CAFA3 timeline, using dataset versions consistent with those used in the challenge. Furthermore, we employed the non‐redundant training and test protein sets provided by the CAFA3 organizers, thereby addressing any concerns regarding data leakage. This careful alignment and use of datasets were crucial in maintaining the integrity of our evaluation process, ensuring that our results are both reliable and comparable to those of other methods assessed in the CAFA3 challenge.

We conducted a comprehensive comparison of our results against 10 top‐performing methods and two baseline approaches (BLAST and Naive) previously documented in the CAFA3 paper. In addition, we incorporated DomFun (Rojano et al., 2022), a domain‐based predictor, and curated InterPro2GO annotations into the evaluation to assess how our models perform relative to other domain‐based prediction methods. DomFun is also designed to predict the functions of domains rather than full proteins. To leverage associations between protein domains and functions, DomFun employs multiple indices based on tripartite network analysis, allowing it to establish relationships between domains and functions. At the protein level, DomFun integrates domain‐function associations to generate accurate protein function predictions. By comparing Domain2GO with DomFun and the other aforementioned methods, we gained valuable insights into the strengths and weaknesses of utilizing domain‐function associations for predicting full protein functions.

We tested two individual sets of protein function predictions for Domain2GO. The first one, “Domain2GO‐S,” was generated via propagating the domain‐GO term mappings that were filtered with an S‐score ≥0.05, to the proteins in the CAFA benchmark dataset. We assigned the co‐occurrence similarity score of the respective domain‐GO term pair as the probabilistic prediction score of the corresponding protein‐GO association prediction. By filtering mappings based on S‐score ≥0.05, we eliminated non‐reliable domain‐GO term mappings and still ended up with a prediction set of reasonable coverage (resulting in 169,534 Domain2GO mappings, which led to 8,695,580 function predictions between 24,665 GO terms and 75,354 CAFA3 target proteins). For the second set, “Domain2GO‐E,” we generated protein‐function predictions this time using Domain2GO mappings previously used as the input of the EM algorithm (78,477 mappings, as explained in Section 2.2) and assigned their E‐value (i.e., the output of the EM algorithm) as the probabilistic scores of the corresponding protein‐GO association predictions. This set contained 2,989,529 predictions between 21,187 GO terms and 73,860 target proteins. We evaluated the results based on the “no‐knowledge benchmarks,” which contain proteins that did not have any experimentally curated annotations in any of the GO ontologies before the benchmark collection period.

We were unable to generate predictions for all target proteins due to the thresholding operations we performed beforehand on domain‐GO mappings and the lack of domain annotation for a part of the dataset. This loss impacted the performance of the proposed method in terms of coverage. The coverage of Domain2GO‐S, Domain2GO‐E and InterPro2GO on the CAFA3 no‐knowledge benchmark set was %0.53, %0.43, and %0.22, respectively. Domain2GO's training data relies on domain‐GO term mappings, which is more limited in terms of dataset size compared to the comprehensive datasets (e.g., readily available amino acid sequences) used by other models. Consequently, it was expected that both Domain2GO‐S and Domain2GO‐E would get lower coverage values than other methods. Despite these limitations, it is worth noting that both Domain2GO‐S and Domain2GO‐E generally achieved higher coverage values in comparison to DomFun. The coverage difference between Domain2GO and DomFun can be attributed to the utilization of different protein‐domain annotation datasets (Rojano et al., 2022). The variations in these datasets are likely significant factors contributing to the observed differences in the predictive capabilities of the two methods. InterPro2GO had the lowest coverage in each GO ontology, which makes sense since it only made predictions based on experimental evidence.

#### 2.5.1 The overall performance evaluation and comparison

**FIGURE 5:**
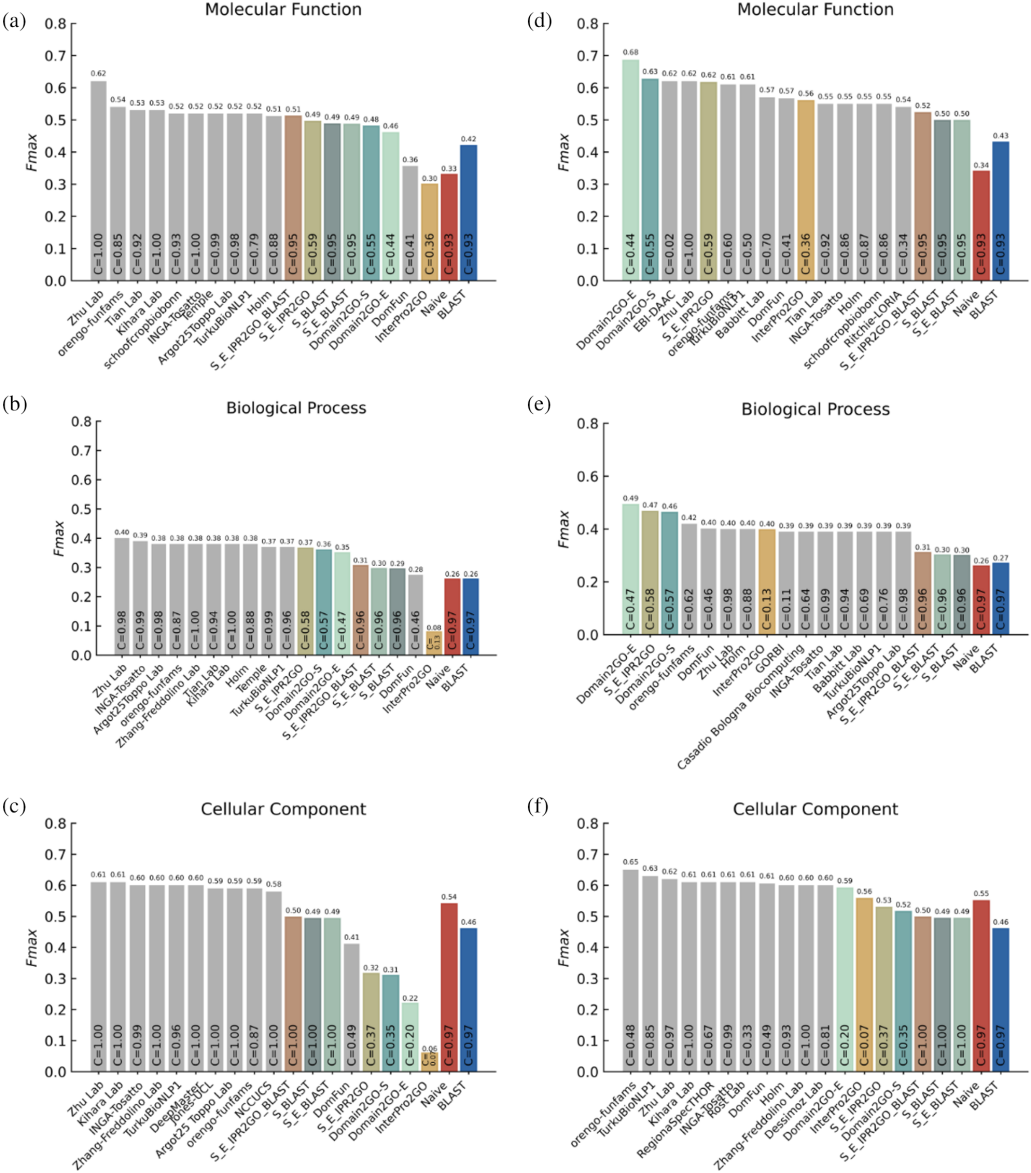
Protein function prediction performance results (*F_max_*) on the CAFA3 benchmark set for three ontologies. The coverage values of all methods, representing the proportion of proteins for which each method provided predictions out of the total number of proteins in the benchmark set, are displayed within the bars. (a–c) *F_max_* bar plots for all methods in the full evaluation mode for the MF, BP, and CC GO categories, respectively. In this mode, prediction methods are assessed based on their performance across all proteins in the benchmark set, regardless of whether they make predictions. (d–f) *F_max_* bar plots for all methods in the partial evaluation mode for the MF, BP, and CC GO categories, respectively. In this mode, prediction methods are evaluated only on the proteins for which they provide predictions. The performance scores of CAFA3 participating models were directly obtained from the challenge article (Zhou et al., 2019). The scores of our models were calculated using the official performance evaluation scripts provided by the CAFA organization (model name abbreviations: S_E_IPR2GO, S_BLAST, S_E_BLAST, and S_E_IPR2GO_BLAST are hybrid approaches combining multiple prediction sets. S_E_IPR2GO: Domain2GO‐S + Domain2GO‐E + InterPro2GO; S_BLAST: Domain2GO‐S + BLAST; S_E_BLAST: Domain2GO‐S + Domain2GO‐E + BLAST; S_E_IPR2GO_BLAST: Domain2GO‐S + Domain2GO‐E + InterPro2GO + BLAST).

Figure 5 shows the performance comparison of Domain2GO with other methods in terms of *F_max_* values. First, our assessment was conducted in the full‐mode, where predictors are assessed on all benchmark proteins regardless of whether they provide predictions (Figure 5a–c). In this evaluation, Domain2GO‐S, Domain2GO‐E and the hybrid models that contain Domain2GO were beaten by the challenge top scorers. The score difference between Domain2GO and the fifth‐best scorer was around %4 (*F_max_*) for the molecular function (MF) category. Whereas the difference between Domain2GO and the third‐best scorer was around %2 (*F_max_*) for the biological process (BP) category (not considering Domain2GO's hybrid models, which had higher performances). Furthermore, Domain2GO‐S and Domain2GO‐E outperformed the baseline predictors and DomFun when predicting MF and BP terms, achieving *F_max_* scores of 0.48 and 0.46 for MFO, and 0.36 and 0.35 for BPO, respectively. However, in predicting cellular component terms, domain‐centric methods, including Domain2GO, encountered difficulties matching the performance of state‐of‐the‐art models.

Subsequently, we conducted an evaluation in the partial‐mode (Figure 5d–f), where prediction methods are only judged on the proteins for which they make predictions. This mode revealed an enhancement in the performance of all domain‐based predictors, including both Domain2GO variants, attributed to their inherently low coverage. In the MFO category, Domain2GO‐E and Domain2GO‐S outperformed all other methods, achieving *F_max_* values of 0.68 and 0.63, respectively. Similarly, in the BPO category, Domain2GO‐E and Domain2GO‐S delivered *F_max_* values of 0.49 and 0.46, surpassing all other methods. This observed performance boost in the partial‐mode highlights a significant challenge in our methodology: while Domain2GO can provide highly accurate predictions, its applicability is constrained by the limited range of proteins it can effectively assess. This limitation becomes particularly evident in the full‐mode evaluation, where the expanded breadth of the assessment encompasses a larger set of proteins.

Another key observation from this evaluation is the contrasting performance of Domain2GO‐S and Domain2GO‐E. While Domain2GO‐S demonstrated greater enrichment in manually curated domain‐GO term mappings (Figure 3) due to its broader coverage, Domain2GO‐E outperformed Domain2GO‐S in the partial‐mode evaluation of the CAFA challenge. This indicates that despite its lower coverage, Domain2GO‐E is capable of offering highly accurate predictions within its narrower prediction range and suggests that it could potentially surpass Domain2GO‐S in predictive performance if not for the high computational requirements that forced us to limit its training dataset. This insight highlights the potential of Domain2GO‐E.

In partial evaluation mode, when predicting cellular component terms, domain‐based models again failed to match the performance of the state‐of‐the‐art models. A few possible reasons for obtaining different performances among the three ontologies could be: (1) differences in the structure of the three ontologies and their indicator data types; (2) the annotation status (completeness) of benchmark proteins; and (3) the biases in the nature of manually curated protein function annotations (Peng et al., 2018). The relatively lower predictive performance of Domain2GO and InterPro2GO in the CCO category could potentially be attributed to the inherent difficulty in associating domains with cellular structures. As domains can be present in various proteins functioning in different cellular locations, establishing accurate domain‐function associations for the CCO category becomes challenging. DomFun employed various statistical measures and domain classification systems (homologous superfamilies or FunFams) to assign association scores to domain‐function associations. They thoroughly evaluated different combinations of classification systems, association, and integration methods to identify the most accurate predictions.

To further validate our findings, we conducted a comparative study between Domain2GO‐S and BLAST, investigating their intersecting and differing true‐positive, false‐positive, and false‐negative predictions at the optimal thresholds (i.e., the threshold where *F_max_* is obtained) for all three ontologies in the full evaluation mode (Figure S8). Even though Domain2GO's low coverage meant that it made fewer true‐positive predictions than BLAST, it also made fewer false‐positive predictions. This underscores Domain2GO‐S's precision and contributes to its overall better performance compared to BLAST in certain aspects. These results emphasize Domain2GO's strength in providing accurate predictions within its defined scope, illustrating its reliability. However, Domain2GO adopts a cautious approach, refraining from making predictions in situations with uncertain or insufficient data. While this approach ensures high reliability, it also indicates that Domain2GO might overlook potential predictions due to its conservative nature. This limitation becomes particularly evident in the full‐mode evaluation, where the tool's effectiveness in broader applications appears reduced.

In general, baseline methods (i.e., Naive and BLAST) are prone to increasing the number of true positives by correctly predicting a small number of relatively generic (shallow) terms, which can be the reason for the competitive performance of baselines (especially Naive method) in the CCO category (Jiang et al., 2016). *S*_min_ scores can provide insight into the capability of a model to make accurate but also specific predictions, as this measure depends on the information content of predicted GO terms. As shown in Table S4, the exceptionally high *F_max_* performance of the Naive method in the CCO category is degraded once the relatively general terms are down‐weighted in the *S_min_*‐based evaluation (lower *S*_min_ values indicate higher performance). In the MFO category, Domain2GO‐S secured the second position with an *S*_min_ value of 6.22, showcasing comparable performance with the top‐performing model, DomFun. Furthermore, in the BPO category, Domain2GO‐S outperformed other domain‐based methods and baseline methods, achieving an *S*_min_ value of 15.93.

#### 2.5.2 Organism‐specific performance comparison

**FIGURE 6:**
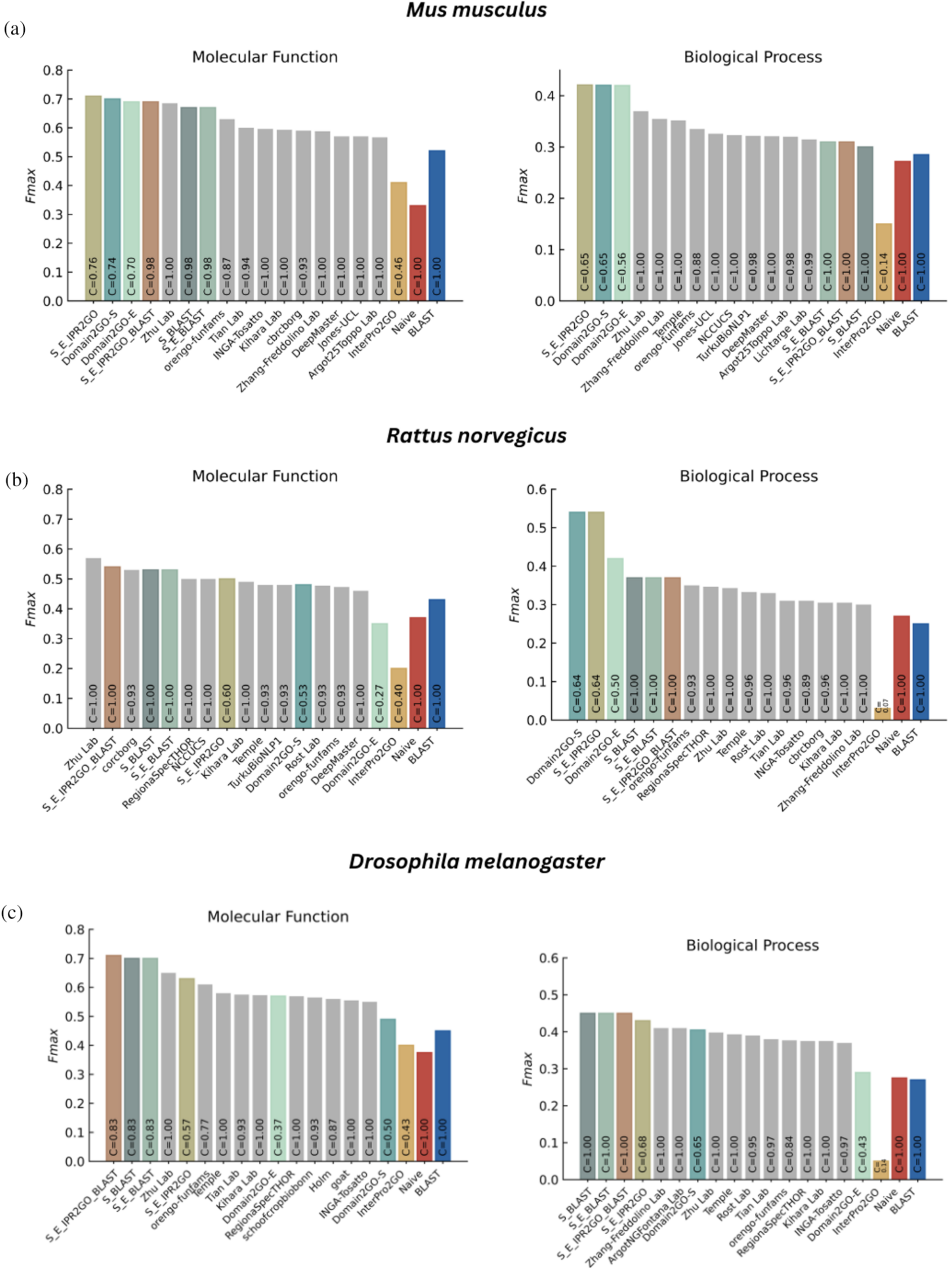
Organism‐specific protein function prediction performance (*F_max_*) in the full evaluation mode. Molecular function and biological process performance results for: (a) *Mus musculus*, (b) *Rattus norvegicus*, and (c) *Drosophila melanogaster*. The performance scores of CAFA3 participating models were directly obtained from the challenge article (Zhou et al., 2019). The scores of our models were calculated using the official performance evaluation scripts provided by the CAFA organization.

The CAFA3 benchmark set encompasses proteins from a wide variety of organisms. To gain insights into the performance of our models when predicting functions across diverse species, we conducted organism‐specific evaluations in the full evaluation mode. The results are displayed in Figure 6 for three selected organisms. Here, we observed that Domain2GO‐S and Domain2GO‐E consistently outperformed all state‐of‐the‐art methods when predicting molecular function and biological process terms for proteins of *Mus musculus*. Our model achieved the *F_max_* values of 0.70 and 0.69 for MFO and 0.42 for both in BPO, respectively (Figure 6a). Similarly, in the case of *Rattus norvegicus*, Domain2GO‐S and Domain2GO‐E exhibited the best performance values in the BPO category with *F_max_* scores of 0.54 and 0.42 and ranked among the top‐performing models in the MFO category with *F_max_* values of 0.48 and 0.35, respectively (Figure 6b). When predicting functions of *Drosophila melanogaster* proteins, Domain2GO‐S and Domain2GO‐E achieved *F_max_* values of 0.49 and 0.57 for MFO and 0.41 and 0.29 for BPO, demonstrating comparable performance with the best‐performing models (Figure 6c). The high performance of our models in predicting the functions of proteins from animal model organisms can be attributed to the extensive and high‐quality domain and functional annotation typically available for model organisms. However, the challenge we faced in the CCO category persisted when evaluating organism‐specific results. A comparison of Domain2GO's performance with other methods concerning five organisms, including the performances in the CCO category, is provided in Supplementary Material, Section 7 and Figures S3–S7.

#### 2.5.3 Evaluation of hybrid prediction approaches

Additionally, we created hybrid protein function prediction approaches that combine multiple prediction sets, including Domain2GO‐S, Domain2GO‐E, InterPro2GO, and BLAST, with the aim of evaluating their collective predictive capabilities. These hybrid methods were established by simply merging their individual prediction sets and assigning the maximum prediction score for predictions that appeared in multiple sets. Among these hybrid approaches, we introduced S_E_IPR2GO, a combination of domain‐based techniques that incorporates Domain2GO‐S, Domain2GO‐E, and InterPro2GO. This approach yielded results very similar to Domain2GO‐S and Domain2GO‐E in both full and partial‐mode evaluations without introducing any significant enhancements in terms of performance or coverage (Figure 5). However, in full‐mode organism‐specific evaluations, especially within the MFO and BPO categories for *Mus musculus*, S_E_IPR2GO demonstrated strong performance. In these assessments, S_E_IPR2GO outperformed Domain2GO‐S and Domain2GO‐E slightly, achieving *F_max_* values of 0.71 and 0.42, making it the top‐performing model (Figure 6a). We also introduced three different combinations of domain‐based models with BLAST: S_BLAST combines Domain2GO‐S with BLAST, S_E_BLAST combines Domain2GO‐S, Domain2GO‐E, and BLAST, while S_E_IPR2GO_BLAST combines Domain2GO‐S, Domain2GO‐E, InterPro2GO, and BLAST. Although the incorporation of an additional prediction model had a positive impact on the performance, with S_E_IPR2GO_BLAST generally exhibiting the best performance among these combinations, these hybrid models did not yield a significant improvement over either the domain‐based models or BLAST alone in the overall performance evaluation (Figure 5). Nevertheless, the inclusion of BLAST proved advantageous for domain‐based models, particularly in enhancing their performance in the cellular component (CCO) category in the full‐mode evaluation (Figure 5c). The potential of hybrid models became more evident in organism‐specific assessments, with S_E_IPR2GO_BLAST achieving second place in the MFO category for *Rattus norvegicus* (Figure 6b), achieving an *F_max_* value of 0.54. S_BLAST and S_E_BLAST followed closely with *F_max_* scores of 0.53. They also demonstrated comparable results to each other, emerging as the top performers in the MFO and BPO categories for *D. melanogaster* (Figure 6c), as well as in the BPO category for *Dictyostelium discoideum* (Figure S6a).

Expanding on our previous analysis of Domain2GO‐S and BLAST's individual performances, outlined in Section 2.5.1 and depicted in Figure S8, we further investigated the impact of their integration, the performance of the Domain2GO‐BLAST method. The high number of mutual predictions between Domain2GO‐S and BLAST across all three ontologies (Figure S8) suggests similar predictive capabilities. This shared predictive strength is crucial for understanding the similar performance levels of the Domain2GO‐BLAST hybrid set when compared individually to Domain2GO‐S and BLAST. Specifically, in the BPO category, the performance of Domain2GO‐BLAST was slightly better than BLAST alone but considerably lower than Domain2GO‐S. This outcome can be attributed to the large number of false‐positive predictions made by BLAST, affecting the combined performance of the hybrid approach.

### 2.6 Availability and usability of protein function prediction methods

Beyond a comparison of predictive performance, it is also equally important to assess methods in terms of usability and interpretability. Highly sophisticated methods, such as the top performers in CAFA3, can indeed be powerful, but they often lack interpretability, making it difficult to grasp the underlying reasons for their specific outputs. Moreover, these methods are often computationally intensive and typically designed and implemented specifically for the challenge, which can limit their practical usability for users beyond the challenge's context. To gain insight into this context, we conducted an investigation of the top performing methods in each CAFA3 category, aiming to assess the availability of user‐friendly tools. The unique number of top performing methods in CAFA3 (including all three GO categories in terms of the *F_max_* metric) was 14. Some of these methods are named after universities and/or research groups; due to this, we were unable to locate the publications or tools for two of them. Among the remaining 12 best‐performing models, only three have currently accessible tools as of November 2023. These include the “Kihara Lab” (Jain & Kihara, 2019) and “Holm” (Törönen & Holm, 2022), both of which provide graphical interfaces, and “schoofcropbiobonn” (Boecker, 2021), which offers a Java‐based program that can be locally installed on a user's computational environment. Apart from those, five of them initially reported in their respective articles that they provided a web server for their method; however, those tools are evaluated to be unavailable due to inaccessible websites or incomplete job processing (despite submitting multiple queries and waiting for extended periods of time for the jobs to be finished). Additionally, four of the methods that lacked a user‐friendly tool made their code available. However, their codebases only included scripts for reproducing the results in their articles and lacked codes dedicated to generating function predictions based on user queries. As observed from this analysis, availability and usability are critical problems in protein function prediction, which indicates the requirement new approaches and tools with practical utility.

Our method, on the other hand, offers several notable advantages for real‐world utility. It provides fine‐resolution functional annotations at the level of protein sequence regions (using the precise annotation of domains), enhancing its interpretability. Additionally, it offers easy comprehensibility and the ability to rapidly generate results, making it highly efficient for working with large datasets. We also developed a user‐friendly tool with a graphical user interface accessible at https://huggingface.co/spaces/HUBioDataLab/Domain2GO. This tool enables users to effortlessly generate function predictions for a query protein sequence in just a few seconds. These characteristics emphasize the practical utility and accessibility of our method, ensuring that our research translates into a valuable and beneficial resource for the broader scientific community.

We conducted a comparative analysis of the best‐performing CAFA3 models with currently available tools in each GO category against Domain2GO‐S and Domain2GO‐E (Table S5). Predictive performance results demonstrated that Domain2GO outscored the best available CAFA3 methods in the MFO and BPO categories in the partial evaluation mode. Moreover, it consistently achieved comparable *F_max_* values to those of the best‐performers for the same GO categories in the full evaluation mode and for the CCO category in the partial evaluation mode.

### 2.7 Generation of the finalized protein function predictions

Finally, we employed final Domain2GO mappings to predict GO term annotations for 566,996 proteins in the UniProtKB/SwissProt database (release 2022_01), by propagating domain‐associated GO terms to the proteins that contain those domains, based on annotations provided in the InterPro database. The resulting protein function prediction set contains 5,046,060 GO term predictions for 291,519 proteins and 11,742 GO terms, which are available at https://github.com/HUBioDataLab/Domain2GO.

## 3 CONCLUSION

In this study, we proposed Domain2GO; a straightforward domain function prediction approach that maps structural domains of proteins to function‐defining GO terms. Two main statistical measures; co‐occurrence similarity and E score, were calculated in order to quantitatively define the association patterns of domains and functions in proteins. An ablation study was performed on manually curated domain function annotations to assess the accuracy of different statistical measures utilized in Domain2GO. Additionally, a literature‐based case study was performed to investigate the significance of Domain2GO mappings.

Even though Domain2GO is not specifically designed to predict protein functions, we looked at how well it did in this area using the CAFA challenge data. The reason behind evaluating the prediction performance of Domain2GO using the task of protein function prediction is the lack of well‐acknowledged benchmarks that are designed for domain function prediction. Our analysis leveraged domain‐GO term mappings to generate protein function predictions, which were then evaluated on the CAFA3 challenge benchmark set. The results demonstrate Domain2GO's strong performance, particularly in the MFO and BPO categories, where it outperforms all other methods in *F_max_* evaluations in the partial evaluation mode, thus emphasizing the robustness of the proposed method. Domain2GO also scored high in the organism‐specific evaluations, particularly in the case of model organisms, consistently ranking as one of the leading models when predicting, again, MFO and BPO terms. Furthermore, we explored simple hybrid function prediction approaches that combine domain‐based methods with sequence‐based BLAST. These approaches brought noteworthy enhancements in the performance of domain‐based models in the prediction of cellular component terms. These findings underscore the advantages of combining diverse data sources and leveraging a broader spectrum of the knowledge space.

It is possible to produce large‐scale GO annotations for unknown proteins using the domain‐GO mappings obtained in this study. In this study, the prediction of GO annotations for 291,519 proteins in the UniProtKB/Swiss‐Prot database only took 4 min on a regular consumer‐level computer. This process can be easily scaled up to the entire UniProtKB (i.e., nearly 249 million protein entries), which is a critical issue for most of the state‐of‐the‐art protein function prediction methods. Furthermore, the web‐based tool we designed for Domain2GO simplifies the function prediction process. This is especially valid for proteins with no prior information available in databases, as the Domain2GO tool solely requires the amino acid sequence of the query protein.

The main limitation we faced in this study was about predicting cellular component terms. This was mainly due to the fact that structural domains are less relevant concerning the localizations of proteins. Additionally, our study highlighted certain limitations when evaluated in the full‐mode of CAFA challenges. In this mode, a general decline in the performance of all domain‐based predictors, including Domain2GO, was observed. This decline is largely attributed to their inherently low coverage, stemming from the limited nature of domain‐based data and the fact that some proteins entirely lack domain annotations, narrowing the scope of predictions achievable through domain‐based approaches (Rojano et al., 2022).

These findings suggest that while Domain2GO is highly reliable in its predictions, offering accurate insights when it does provide a prediction, its performance is constrained when evaluated across a broader range. In the field of predictive modeling, using a single type of input data may provide a limited perspective regarding the task at hand. In this sense, a potential area of exploration could be extending the Domain2GO framework by incorporating various complementary protein centric data sources. Furthermore, the biological entity association approach proposed in this study can be modified with the use of additional types of features and ontologies from integrative data sources such as heterogeneous biological knowledge graphs (Doğan et al., 2021) in order to develop novel multi‐modal systems that can predict unknown biomolecular relationships.

Previous research such as that conducted by Bashton and Chothia (2007) has shown that domain functions can change depending on the context, especially within multi‐domain architectures. Our methodology evaluates each domain independently, which may not fully capture context‐dependent nuances. To overcome this limitation, future research could focus on developing an enhanced methodology capable of effectively predicting context‐dependent functions via leveraging mappings between pairs or triplets of domains and GO terms. Such advancements would not only enhance the accuracy of Domain2GO, but also broaden its applicability to encompass the diverse functional landscape. By pursuing this direction, we aim to contribute a more comprehensive and versatile tool for function prediction, deepening the understanding of the dynamic nature of protein functionality.

## 4 MATERIALS AND METHODS

The overview of the proposed methodology is shown in Figure 1. First, we prepared the domain and GO annotation datasets and used the UniProt accessions in both sets to map InterPro domains to corresponding GO terms (Figure 1a,b). We identified each pair as a(n) initial/raw Domain2GO mapping. The training part of the system consists of two statistical score calculation steps. First, we calculated a co‐occurrence similarity measure (*S*) for each Domain2GO mapping. This measure was utilized in statistical resampling (via the Kolmogorov–Smirnov test), which is employed to differentiate significant mappings from the ones that occurred by chance (Figure 1c,d). Second, we applied the expectation maximization (EM) algorithm (Dempster et al., 1977), which is a method for obtaining a maximum‐likelihood estimate of the probability of association (*θ*), and the confidence of its probability (*E*), independently for each Domain2GO mapping. All Domain2GO mappings were ranked independently by four of the calculated measures (i.e., *S*, *θ*_*i*_, *θ*, and *E*) and the enrichment of manually curated Domain2GO annotations in the highest‐ranking mappings was analyzed. The initial mappings were filtered using the thresholds established in previous steps, and the resulting mappings were employed to generate protein function predictions (Figure 1e).

### 4.1 Dataset

In order to generate domain‐function mappings, we combined domain‐protein and protein‐function associations. For the domain‐protein annotation dataset, the UniProtKB‐InterPro entry annotation file was downloaded from the InterPro database version 85.0. This file contained 615,060,473 annotations between 38,913 InterPro entries of all types and 179,520,976 UniProtKB proteins. After eliminating all InterPro entry types other than domains, the final version of the file contained 222,753,705 annotations between 11,233 InterPro domain entries and 121,492,444 UniProtKB proteins.

The second dataset contained protein function annotations, which were obtained by following a procedure utilized in (Doğan et al., 2016), as follows: GO annotations with manual and experimental evidence codes (i.e., all except electronic annotations—IEA, due to their questionable reliability) were downloaded from the UniProt‐GOA database version 193 (Huntley et al., 2015) for the protein entries in the UniProtKB/Swiss‐Prot database (UniProt Consortium, 2023). The GO terms in the dataset were propagated to their parent terms based on their “is a” and “part of” relationships to enrich the annotations. In its final form, the dataset had 6,998,018 annotations between 125,309 proteins and 30,959 GO terms.

### 4.2 Calculating the co‐occurrence similarities

We used the UniProt IDs as the key to connect two datasets and generate domain‐GO term mappings. If a UniProt ID is shared between (co‐annotated with) an individual domain and a GO term, we identify this pair as a(n) initial/raw Domain2GO mapping. This way, 2,069,796 mappings were obtained between 8642 domains and 28,420 GO IDs. In the first step of the training procedure, a filtering operation was performed to eliminate mappings resulting from the coincidental occurrence of a domain and GO ID on the same protein. For this, we calculated a co‐occurrence similarity score (Equation 1.), inspired by an information‐theoretic definition of similarity (Lin, 1998) for each domain‐GO term mapping.

(1) $$S_{\mathrm{Di},\mathrm{GOj}}=\frac{2*N_{\mathrm{Di},\mathrm{GOj}}}{\;N_{\mathrm{Di}\;}+N_{\mathrm{GOj}}},$$

where *S*_Di,GOj_ is the co‐occurrence similarity of a domain *i* and GO term *j* pair, *N*_Di,GOj_ is the number of proteins that are annotated with both “domain *i*” and “GO term *j*,” *N*_Di_ is the total number of proteins with the annotation “domain *i*” and *N*_GOj_ is the total number of proteins with the annotation “GO term *j*.”

An example of the co‐occurrence similarity calculation is shown in Figure 1b,c. A high degree of co‐occurrence similarity indicates that the number of proteins to which a protein domain and GO terms are co‐annotated is significantly greater than the total number of proteins to which these terms are individually annotated. Therefore, a threshold co‐occurrence similarity value can be set to differentiate the relevant Domain2GO pairs from the ones that occurred by chance. Assuming that some pairs with a high similarity score can still be random, especially when *N*_Di_ and *N*_GOj_ are very low, we employed *N*_Di,GOj_ as a second parameter abbreviated as “*n*.” Accordingly, Domain2GO pairs with both high *S* and *n* values are considered to be more reliable.

In order to assign optimal *S* and *n* values to be used as thresholds, we compared the co‐occurrence similarity distribution obtained from Domain2GO mappings to the score distribution of randomly generated domain‐GO term pairs. The idea behind comparing these distributions is that the frequency of mappings with high and low *S* values should differ between the original and random mapping sets. For this, a randomized mapping table was created by shuffling domain and GO term columns on the original Domain2GO mappings (Figure 1c), and then the *S* and *n* score calculation steps were repeated for the newly generated random Domain2GO mappings.

Thirty candidate threshold value combinations were generated for *S* (i.e., *S* < 0.1, <0.2, <0.3, <0.4, <0.5, and <0.6) and *n* (*n* ≥1, ≥2, ≥3, ≥4, and ≥5). The co‐occurrence similarity distribution histograms of the mappings with *S* and *n* values higher than the candidate thresholds were plotted for both the original and randomized mapping sets. As a last step, we employed a widely used non‐parametric test, Kolmogorov–Smirnov (KS) (Hollander et al., 2013; Lilliefors, 1967), which compares two samples and estimates whether they are drawn from the same distribution by quantifying the distance between the samples. The null hypothesis states that the samples are drawn from the same distribution. The KS‐test was applied to 30 different co‐occurrence similarity distributions, which were plotted for each of the candidate threshold value combinations. The minimum value pair that is able to show that the original and random mapping sets are drawn from significantly different distributions (i.e., the lowest values the null hypothesis is rejected at) was selected as the official thresholds.

### 4.3 Expectation maximization

#### 4.3.1 Estimating association probabilities of Domain2GO pairs

For the second step of the training process, we applied the EM algorithm (Dempster et al., 1977) which was implemented in earlier studies for different prediction purposes, such as domain‐domain interaction prediction (Deng et al., 2002; Riley et al., 2005). Figure S1 depicts a toy example of EM algorithm implementation on the Domain2GO mapping set.

The algorithm was initialized by obtaining an estimate of the association probability for each Domain2GO mapping, based on the ratio of proteins they are co‐annotated to. In order to do this, all the domain *i*‐protein‐GO term *j* triplets defined by the co‐annotation of the domain *i* and a GO term j on any protein were represented with an association value which is denoted as $C_{p}^{\mathrm{Di},\;\mathrm{GOj}}$ which is updated as EM algorithm proceeds. All $C_{p}^{\mathrm{Di},\;\mathrm{GOj}}$ values were initialized by setting them to 1 (Equation 2), assuming that all possible domain‐GO term pairs are associated regardless of the number of proteins they are co‐annotated to. Based on $C_{p}^{\mathrm{Di},\;\mathrm{GOj}}$ values, two variables; *M*_Di,GOj_ and *K*_Di,GOj_ were defined for each domain‐GO term pair.

(2) $$C_{p}^{\mathrm{Di},\;\mathrm{GOj}}=\{\begin{array}{l} 1\;\text{if}\;\mathrm{GO}\;\text{term}\;j\;\text{and domain}\;i,\text{which}\;\mathrm{are}\;\text{both annotated to protein}\;p,\mathrm{are}\;\text{associated}\\ 0\;\text{otherwise, } \end{array}$$

(3) $$M_{\mathrm{Di},\;\mathrm{GOj}}=\sum_{p}C_{p}^{\mathrm{Di},\mathrm{GOj}},$$

(4) $$K_{\mathrm{Di},\;\mathrm{GOj}}=\sum_{p}\left(1-C_{p}^{\mathrm{Di},\mathrm{GOj}}\right).$$

*M*_Di,GOj_ was initialized with the number of proteins that are annotated with both domain *i* and GO term j are annotated together and *K*_Di,GOj_ was initialized with zero based on their equations, as all initial $C_{p}^{\mathrm{Di},\;\mathrm{GOj}}$= 1. Finally, *Z*_Di,GOj_ is defined, which is the total unique protein count where Di is annotated to, but GOj is not annotated to and where GOj is annotated to, but Di is not annotated to.

After these initial measures are defined, we calculated the initial estimate of the θ_Di,GOj_; the probability that Di and GOj are associated (Equation 5).

(5) $$\theta_{\mathrm{Di},\mathrm{GOj}}^{\text{init}}=\frac{M_{\mathrm{Di},\mathrm{GOj}}}{M_{\mathrm{Di},\mathrm{GOj}}+K_{\mathrm{Di},\mathrm{GOj}}+Z_{\mathrm{Di},\mathrm{GOj}}\;}.$$

Initialized values of θ_Di,GOj_ and other variables for all domain‐GO term pairs were then used to estimate the initial likelihood L (Equation 6) of the Domain2GO mapping set:

(6) $$L=\prod_{\mathrm{Di},\mathrm{GOj}}{\theta_{\mathrm{Di},\mathrm{GOj}}}^{M_{\mathrm{Di},\mathrm{GOj}}+\alpha }{\left(1-\theta_{\mathrm{Di},\mathrm{GOj}}\right)}^{K_{\mathrm{Di},\mathrm{GOj}}+Z_{\mathrm{Di},\mathrm{GOj}}+\beta },$$

where α and β are arbitrary pseudocounts to ensure that obtaining too high or too low θ_Di,GOj_ values were not due to the low frequencies of Di and GOj annotations, but to the large numbers of co‐occurrences indicating the potential association of them. Di, GOj is each domain‐GO term pair in Domain2GO mapping set.

After initialization, we followed four basic steps of the EM algorithm (Figure S1a):

1. The updated value of $C_{p}^{\mathrm{Di},\;\mathrm{GOj}}$ was calculated for each triplet based on the of θ_Di,GOj_ calculated in previous iteration (based on the initial estimate of θ_Di,GOj_ (i.e., $\theta_{\mathrm{Di},\mathrm{GOj}}^{\text{init}}$) in the first iteration):
(7) $$\;U\left[C_{p}^{\mathrm{Di},\;\mathrm{GOj}}\right]=\frac{\theta_{\mathrm{Di},\;\mathrm{GOj}}}{1-\prod_{\mathrm{Dx}\;\in A\left(p\right),\mathrm{GOy}\in A\left(p\right)}\left(1-\theta_{\mathrm{Dx},\mathrm{GOy}}\right)},$$
  where Dx and GOy are among the annotations of the protein *p* (i.e., A(p)) in Di‐p‐GOj triplet and Di, GOj is the association probability for Di‐GOj pair which was calculated at the end of the previous iteration.
2. M_Di,GOj_ and K_Di,GOj_ were recalculated (Equation 3 and 4) for each domain‐GO term pair from the updated value of $C_{p}^{\mathrm{Di},\;\mathrm{GOj}}$.
3. θ_Di,GOj_ was re‐estimated (Equation 5) from the updated values of *M*_Di,GOj_ and *K*_Di,GOj_.
4 Likelihood was re‐estimated (Equation 6).

We repeated these steps until the likelihood (Equation 6) stops increasing noticeably. The probability matrix of all Domain2GO domains, *θ*, was obtained as the result of the EM algorithm. A high θ_Di,GOj_ value shows that the mapped domain and GO term have a high probability of being associated. At this point, the issue mentioned in Section 4.2 emerges again, due to the possibility of observing pairs with high *θ* just by chance when some domains and GO terms were annotated to a high number of proteins. Furthermore, a domain‐GO term mapping, in which the corresponding GO term is highly specific, may have a low *θ* just because the domain and GO term are both annotated to a low number of proteins. A likelihood ratio test was performed in order to differentiate highly significant domain‐GO term mappings from the ones that could be observed just by chance.

#### 4.3.2 Computing E scores (likelihood ratio test)

A new probability matrix, $\bar{\theta }$ ^Di,GOj^, was defined by running the EM algorithm again for each Di, GOj pair, under the model that ${\bar{\theta }}_{\mathrm{Di},\mathrm{GOj}}^{\mathrm{Di},\mathrm{GOj}}$ is set to zero at the first step of every iteration. This resulted in a maximum likelihood estimate of all Domain2GO mappings excluding Di‐GOj pair. A measure of evidence, *E*_Di,GOj_, is calculated by using the $\bar{\theta }$ ^Di,GOj^ matrix defined specifically for each pair (Figure S1b):

(8) $$E_{\mathrm{Di},\;\mathrm{GOj}}=\sum_{p:\;\mathrm{Di}\;\in A\left(p\right),\mathrm{GOj}\in A\left(p\right)}\log\frac{1-\prod_{\mathrm{Dx}\;\in A\left(p\right),\mathrm{GOy}\in A\left(p\right)}1-\theta_{\mathrm{Dx},\mathrm{GOy}}}{1-\prod_{\mathrm{Dx}\;\in A\left(p\right),\mathrm{GOy}\in A\left(p\right)}1-{\bar{\theta }}_{\mathrm{Dx},\mathrm{GOy}}^{\mathrm{Di},\mathrm{GOj}}},$$

where *p* is the protein set that is co‐annotated with the given Di‐GOj pair, Dx and GOy are among the annotations of a protein in the protein set *p* (i.e., *A*(*p*)), *θ* is the probability matrix that is calculated in Section 4.3.1 for all domain‐GO term mappings, $\theta_{\mathrm{Dx},\mathrm{GOy}}$ is the θ probability matrix value of domain‐GO term pairs that are co‐annotated to a protein in the protein set *p*, $\bar{\theta }$ ^Di,GOj^ is the probability matrix of all domain‐GO term mappings under the model that the probability of the given Di‐GOj mapping (${\bar{\theta }}_{\mathrm{Di},\mathrm{GOj}}^{\mathrm{Di},\mathrm{GOj}}$) is equal to zero and ${\bar{\theta }}_{\mathrm{Dx},\mathrm{GOy}}^{\mathrm{Di},\mathrm{GOj}}$ is the $\bar{\theta }$ ^Di,GOj^ probability matrix value of domain‐GO term pairs that are co‐annotated to a protein in the protein set *p*.

While *θ* can be considered as the probability of a mapping, the *E* score aims to detect highly specific Domain2GO mappings with extensive evidence in the co‐annotation data. The number of instances of a mapping should increase the confidence in that annotation; therefore, a high *E*_Di,GOj_ is expected for a pair with high *θ*. More importantly, a pair with low *θ* can also have a high *E*_Di,GOj_ if θ_Di,GOj_ is relatively high in comparison with the competing ${\bar{\theta }}_{\mathrm{Di},\mathrm{GOj}}^{\mathrm{Di},\mathrm{GOj}}$ value which is calculated by taking only the competing domain‐GO term pairs (pairs that are co‐annotated to the same protein set other than Di, GOj) into account. On the contrary, a low *E*_Di,GOj_ implies that this pair is not any better at explaining co‐annotations than competing hypotheses (other potential pairs which are co‐annotated to the same protein).

### 4.4 Competing function prediction methods

Two baseline methods, Naive and BLAST, were built in order to compete with the co‐occurrence similarity and E score‐based function predictions of Domain2GO. The Naive method simply pairs each input protein with all existing GO terms and assigns the GO term's annotation frequency in the source database as the prediction score for all pairs containing that GO term. Both of the baseline methods were constructed as explained in the CAFA experiments (Zhou et al., 2019), using the Swiss‐Prot annotations that existed before the CAFA3 annotation collection period (date: 2016/09). In addition to these baseline methods, we included the performance scores of top‐performing models in the CAFA3 challenge as part of our evaluation.

InterPro2GO annotations were included as another competing model that considers domain‐based knowledge. It is possible that the current InterPro2GO release contains the annotations obtained within the CAFA3 benchmark collection period. In order to avoid giving InterPro2GO an unfair advantage, we used the September 2016 InterPro2GO release. Domain‐GO term pairs from the InterPro2GO data were employed to generate protein‐function predictions on the CAFA3 target protein set. As InterPro2GO annotations are created through manual curation, all prediction scores were set to 1. Another important thing is that only annotations produced during the annotation collection period are used to generate the CAFA benchmark set. Hence, a prediction may not be included in the CAFA benchmark dataset even though it is verified after the end of the challenge. Therefore, these predictions are evaluated as false positives during evaluation even though they are manually curated.

In addition, DomFun (Rojano et al., 2022) was included as another competing method. DomFun adopts a similar approach to Domain2GO, predicting domain functions through protein domain associations. It utilizes tripartite network analysis with multiple indices to establish robust domain‐function connections. For protein function prediction, DomFun identifies constituent domains of a given protein within the CATH‐Gene3D database (Sillitoe et al., 2015) and then seeks associated functions. When a protein contains multiple domains linked to the same functional annotation, DomFun integrates the association values into a single combined score.

### 4.5 Performance evaluation metrics

We employed the CAFA3 challenge (Zhou et al., 2019) benchmark set to investigate whether Domain2GO can be extended to predict protein functions, even though it is primarily proposed to predict the functions of domains.

To facilitate a fair comparison, we recalibrated the Domain2GO mappings to be in sync with the specific timeframe of the CAFA3 experiment. This involved using data from September 2016, chosen to correspond with the dataset versions employed by other participating methods in the CAFA3 challenge. After recalculating these mappings, we applied them to generate functional predictions for the proteins within the CAFA3 target set. These predictions were subsequently assessed against the benchmarks established in the CAFA3 experiment, thereby allowing us to evaluate the efficacy and accuracy of Domain2GO in the context of a recognized and rigorous protein function prediction challenge. Here, we used the protein‐centric evaluation mode, which measures the accuracy of assigning GO terms to a protein by calculating two basic evaluation metrics: *F_max_* and minimum semantic distance (*S*_*min*_) (Equation 9 and 12).

*F*_*max*_ (Equation 9) corresponds to the maximum of the *F*‐scores that are computed using precision (Equation 10) and recall (Equation 11) values at each prediction score threshold (*τ*).

(9) $$F_{\max}=\max_{\tau }\left\{\frac{2*\mathrm{pr}\left(\tau \right)*\mathrm{rc}\left(\tau \right)}{\mathrm{pr}\left(\tau \right)+\mathrm{rc}\left(\tau \right)}\right\},$$

(10) $$\mathrm{pr}\left(\tau \right)=\frac{{\mathrm{TP}}_{\tau }}{{\mathrm{TP}}_{\tau }+{\mathrm{FN}}_{\tau }},$$

(11) $$\mathrm{rc}\left(\tau \right)=\frac{{\mathrm{TP}}_{\tau }}{{\mathrm{TP}}_{\tau }+{\mathrm{FP}}_{\tau }.}$$

where *TP*τ, *FN*τ, and *FP*τ represent the number of true positives, false negatives and false positives at the prediction score threshold *τ*.

*S*_min_ corresponds to the minimum semantic distance between real and predicted annotations computed using remaining uncertainty (ru) and misinformation (mi) values over all prediction score thresholds. *S*_min_ (Equation 12) evaluates the predictive performance by taking into account the information content of GO terms, down‐weighing the less informative annotations as they are not as valuable.

(12) $$S_{\min}=\min_{\tau }\left\{\sqrt{\mathrm{ru}{\left(\tau \right)}^{2}+\mathrm{mi}{\left(\tau \right)}^{2}}\right\}.$$

Detailed information regarding the definition and calculation of these metrics can be found in Jiang et al. (2016). The prediction score thresholds are selected by the CAFA3 evaluation protocol to generate binary predictions from submitted continuous prediction scores. Additionally, we report a secondary metric, coverage, corresponding to the percentage of the proteins in the benchmark that are predicted by the proposed method.

Co‐occurrence similarity and *E* score results were evaluated as two distinct models to determine their predictive capabilities. To calculate the performance using the CAFA3 protocol, prediction scores must be within the range of (0.00, 1.00]. For the first prediction score set, we directly used the co‐occurrence similarity scores (which were already set to the required range) calculated in Equation (1). If a protein with an existing domain *i* annotation receives the GO term *j* prediction, then this protein‐function pair obtains the prediction score *S*_Di,GOj_. For the second prediction score set, we used the *E* scores calculated in Equation (8), after normalization by min‐max scaling. Similar to the first one, if a protein with domain *i* annotation receives the GO term j prediction, then this protein‐function pair receives the normalized *E*_Di,GOj_ value as its prediction score.

## AUTHOR CONTRIBUTIONS

**Tunca Doğan:** Conceptualization; investigation; writing – original draft; methodology; validation; visualization; writing – review and editing; supervision; formal analysis; project administration. **Erva Ulusoy:** Investigation; writing – original draft; methodology; validation; visualization; writing – review and editing; software; data curation.

## Supporting information


**DATA S1:** Supporting Information.

## Data Availability

Domain2GO is shared as an open access programmatic tool, along with all datasets used and results generated, at https://github.com/HUBioDataLab/Domain2GO. It is also offered as an online web‐based tool with a graphical user interface at https://huggingface.co/spaces/HUBioDataLab/Domain2GO.
