# Supplementary material for: Mutual annotation‐based prediction of protein domain functions with Domain2GO
Source: Protein Sci. 2024 May 16;33(6):e4988. doi: 10.1002/pro.4988 (PMC11099699; doi:10.1002/pro.4988)
Supplement: Supplementary file 1 — DATA S1: Supporting Information. [file PRO-33-e4988-s001.pdf]

Supplementary Material

Mutual Annotation-Based Prediction of Protein Domain Functions with Domain2GO

Erva Ulusoy[1,2], Tunca Doğan[1,2,*]

[1] Biological Data Science Lab, Department of Computer Engineering, Hacettepe University, Ankara, Turkey

[2] Department of Bioinformatics, Graduate School of Health Sciences, Hacettepe University, Ankara, Turkey

*To whom correspondence should be addressed: tuncadogan@gmail.com

1. Schematic representation of expectation maximization (EM) algorithm

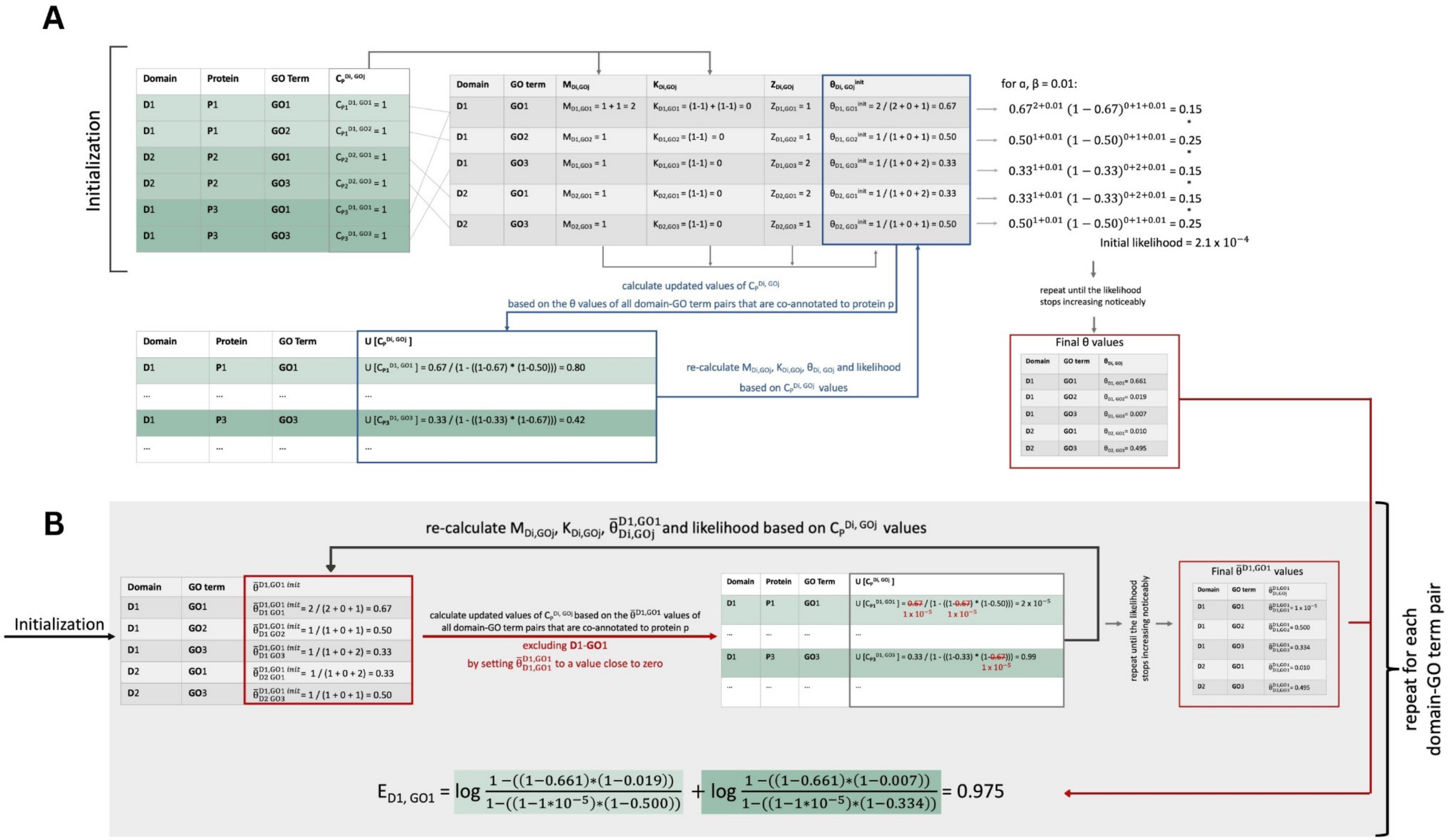

Figure S1. Schematic representation of expectation maximization (EM) algorithm on a toy example. (A) Estimating association probabilities of Domain2GO pairs. $C_p^{Di, GOj}$ values were defined for each domain-protein-GO term triplet and $M_{Di, GOj}$, $K_{Di, GOj}$, $Z_{Di, GOj}$, $\theta_{Di, GOj}$ values were calculated for each domain-GO term pair based on $C_p^{Di, GOj}$ values. $C_p^{Di, GOj}$ values were updated based on the $\theta_{Di, GOj}$ values of the previous iteration, and $M_{Di, GOj}$, $K_{Di, GOj}$, $\theta_{Di, GOj}$ values were updated based on updated $C_p^{Di, GOj}$ values. These steps were iterated until the likelihood value, calculated based on $\theta_{Di, GOj}$ values at the end of each iteration, reached a stopping point. **(B) Computing the evidence -E- score of the D1-GO1 pair ($E_{D1, GO1}$).** $C_p^{Di, GOj}$, $M_{Di, GOj}$, $K_{Di, GOj}$, $Z_{Di, GOj}$ and $\underline{\theta}^{D1,GO1}_{Di,GOj}$ values were initialized as shown in panel A. Updated $C_p^{Di, GOj}$ values were calculated based on the $\underline{\theta}^{D1,GO1}_{Di,GOj}$ values of the previous iteration, excluding D1-GO1 pair from competing pairs (taking only D1-GO2 on P1 and D1-GO3 on P3 into account) by setting $\underline{\theta}^{D1,GO1}_{D1,GO1}$ to a value close to zero. $M_{Di, GOj}$, $K_{Di, GOj}$ and $\underline{\theta}^{D1,GO1}_{Di,GOj}$ values were updated based on updated $C_p^{Di, GOj}$ values. These steps are iterated as explained in panel A. Finally, $E_{D1, GO1}$ value was calculated based on final $\theta_{Di, GOj}$ values that are calculated in panel A and $\underline{\theta}^{D1,GO1}_{Di,GOj}$ values that are calculated in panel B.

2. Initial Domain2GO mapping statistics at different co-occurrence similarity thresholds

The table below displays the numbers of Domain2GO pairs, domains, and GO terms in the original and randomized mapping sets at different co-occurrence similarity thresholds. It is observed that the original and randomized mapping sets have a similar number of mappings when the S threshold is lower. However, the gap between them widens when we look at higher S thresholds. This is expected given that a domain i and a GO term j are much less likely to co-occur on the same protein when their indices are randomized. This also explains the lower number of domains and GO terms at higher S values for the randomized mapping set.

Table S1. Statistics of the initial original and randomized Domain2GO mappings (n≥1 for all values).

Threshold (S)	# of mappings		# of unique domains		# of unique GO terms	
	Original mapping	Random mapping	Original mapping	Random mapping	Original mapping	Random mapping
= 1	470	35	319	35	350	34
≥ 0.9	902	35	574	35	679	34
≥ 0.8	1,667	35	997	35	1,211	34
≥ 0.7	2,511	35	1,359	35	1,752	34
≥ 0.6	4,278	212	2,076	207	2,847	195
≥ 0.5	6,837	570	2,917	542	4,398	479
≥ 0.4	10,619	1,096	3,741	1,016	6,392	832
≥ 0.3	16,801	1,811	4,602	1,622	9,122	1,240
≥ 0.2	32,028	6,626	5,775	2,986	14,236	4,929
≥ 0.1	78,477	30,459	6,870	5,509	21,466	12,692
≥ 0.0	2,069,796	4,584,047	8,642	8,642	28,420	28,420

3. KS test significance results for comparing original vs. randomized co-occurrence similarity distributions

Table S2. KS test significance results for the comparison of original vs. randomized co-occurrence similarity distributions at different co-occurrence similarity (S) and the number of co-annotated proteins (n) thresholds. Each cell includes the count of domain-GO term mappings for the respective sample. The statistical significance is denoted by symbols next to the counts: 'ns' indicates not significant for $p > 0.05$, a single asterisk (*) signifies significance for $p \leq 0.01$, double asterisks (**) denote high significance for $p \leq 0.0001$, and triple asterisks (***) represent the highest significance for $p \leq 0.00001$.

	**S > 0.1**	**S > 0.2**	**S > 0.3**	**S >0.4**	**S > 0.5**	**S > 0.6**
**n >= 1**	76,468 ns	30,472 ns	16,545 (**)	9,545 (***)	5,944 (***)	4,138 (***)
**n >= 2**	63,385 (*)	26,696 (***)	14,775 (***)	8,694 (***)	5,475 (***)	3,669 (***)
**n >= 3**	50,301 (**)	22,415 (***)	12,730 (***)	7,750 (***)	4,972 (***)	3,328 (***)
**n >= 4**	41,606 (**)	19,387 (***)	11,283 (***)	6,985 (***)	4,524 (***)	3,066 (***)
**n >= 5**	36,823 (**)	17,425 (***)	10,289 (***)	6,425 (***)	4,195 (***)	2,835 (***)

4. Statistical measures of selected Domain2GO mappings

Table S3. Arbitrarily selected high-confidence domain-GO term pairs ranked by their S score. Pairs that are manually associated in the InterPro2GO set are marked with a "✓". The last 3 rows include the pairs that are examined as case studies to assess their biological relevance (see Section 2.4).

**InterPro ID**	**GO ID**	**θ_i**	**θ**	**E**	**n**	**S**	**Are the domain & GO term manually associated as well?**
IPR000426	GO:0019773	0.022	0.431	7.783	127	1	✓
IPR039430	GO:0046077	0.077	0.055	0.502	44	1	
IPR020602	GO:0003934	0.019	0.171	1.448	40	1	
IPR000965	GO:0004350	0.008	0.276	17.484	39	1	✓
IPR011095	GO:0008716	0.006	0.176	2.15	36	1	✓
IPR003526	GO:0008685	0.006	0.496	33.076	31	1	✓

IPR032678	GO:0006423	0.029	0.275	1.13	59	0.992	
IPR022630	GO:0004478	0.143	0.208	0.69	57	0.991	✓
IPR020598	GO:0000179	0.028	0.401	3.215	56	0.991	✓
IPR033828	GO:0003883	0.031	0.067	0.807	52	0.99	✓
IPR033705	GO:0006438	0.077	0.253	1.515	50	0.99	
IPR039430	GO:0046072	0.077	0.055	0.502	44	0.989	
IPR022417	GO:0004418	0.017	0.294	3.332	43	0.989	✓
IPR035490	GO:0004360	0.008	0.087	1.301	42	0.988	
IPR033644	GO:0004325	0.018	0.302	3.106	39	0.987	
IPR013840	GO:0003911	0.03	0.068	0.771	38	0.987	✓
IPR039429	GO:0004372	0.008	0.059	1.123	69	0.986	
IPR013512	GO:0051483	0.002	0.055	0.401	33	0.985	
IPR031481	GO:0046920	0.003	0.394	4.066	33	0.985	
IPR018149	GO:0004824	0.125	0.094	2.311	49	0.98	✓
IPR033731	GO:0004820	0.167	0.438	50.044	72	0.98	
IPR012310	GO:0003910	0.031	0.129	2.273	46	0.979	✓
IPR004971	GO:0004482	0.182	0.403	7.345	21	0.977	
IPR020630	GO:0004486	0.042	0.097	1.513	60	0.976	
IPR033659	GO:0004325	0.018	0.195	1.093	38	0.974	
IPR011128	GO:0047952	0.017	0.24	2.728	55	0.973	
IPR022663	GO:0008839	0.016	0.154	0.779	33	0.971	✓
IPR041739	GO:0004349	0.008	0.231	6.538	31	0.969	
IPR004255	GO:0004144	0.016	0.097	0.436	15	0.968	✓
IPR005110	GO:0042040	0.003	0.081	0.266	15	0.968	
IPR001442	GO:0005587	0.022	0.149	0.612	15	0.968	

IPR005110	GO:0018315	0.045	0.081	0.266	15	0.968	
IPR007471	GO:0004057	0.167	0.18	0.561	15	0.968	✓
IPR039430	GO:0009189	0.015	0.055	0.502	44	0.967	
IPR015341	GO:0004559	0.016	0.151	1.435	29	0.967	✓
IPR035490	GO:0070548	0.033	0.087	1.301	42	0.966	
IPR011258	GO:0004619	0.005	0.242	1.469	28	0.966	✓
IPR009721	GO:0047196	0.083	0.089	0.346	14	0.966	
IPR022673	GO:0004340	0.008	0.065	0.611	41	0.965	
IPR020602	GO:0006729	0.004	0.171	1.448	40	0.964	
IPR041711	GO:0019988	0.023	0.093	0.914	39	0.963	
IPR005849	GO:0008108	0.027	0.21	0.62	13	0.963	✓
IPR018164	GO:0004813	0.029	0.066	1.284	63	0.962	✓
IPR022648	GO:0044796	0.007	0.127	0.72	25	0.962	
IPR035476	GO:0004347	0.008	0.118	1.813	49	0.961	
IPR013548	GO:0002116	0.006	0.051	0.277	24	0.96	
IPR000070	GO:0030599	0.005	0.345	23.678	69	0.958	✓
IPR001017	GO:0004591	0.005	0.035	0.325	22	0.557	
IPR001678	GO:0008169	0.03	0.017	0.164	24	0.387	
IPR003583	GO:0051103	0.25	0.015	0.166	25	0.394	

5. Biological relevance of the selected mappings—a case study (Case 2-3)

The second case is a Domain2GO mapping between the domain “dehydrogenase, E1 component” (IPR001017) and the GO molecular function term “oxoglutarate dehydrogenase (succinyl-transferring) activity” (GO:0004591). These two terms were mapped to each other with a co-occurrence similarity (S) value of 0.56, on a total of 22 proteins (n). According to the InterPro database, IPR001017 domain is found in multiple proteins that have dehydrogenase activity, one of which is the 2-oxoglutarate dehydrogenase (EC 1.2.4.2). (URL:https://www.ebi.ac.uk/interpro/entry/InterPro/IPR001017/) The E1 subunit catalyzes the decarboxylation of this enzyme by transferring a succinyl group onto the lipoyl-group bound to the E2 subunit (Tretter and Adam-Vizi 2005). Clearly, this domain is in charge of the molecular function defined by the Domain2GO mapped GO term. There is an InterPro2GO generated association between IPR001017 and the molecular function term “oxidoreductase activity, acting on the aldehyde or oxo group of donors, disulfide as acceptor” (GO:0016624), which is related (i.e., with is_a relationship) to the Domain2GO predicted GO:0004591 as its parent term. Additionally, Domain2GO captured another association between IPR001017 and the cellular component term “oxoglutarate dehydrogenase complex” (GO:0045252), which is a multienzyme complex that contains 2-oxoglutarate dehydrogenase as one of its main components (Bunik and Degtyarev 2008). GO:0045252 is also related to GO:0004591 (i.e., with capable_of relationship). This example shows that Domain2GO was able to associate IPR001017 with GO terms that are more specific compared to the manually curated GO association. In addition to that, we found additional associations between this domain and different cellular component terms, each of which is one-step away from the above-mentioned terms (and from each other) on the GO DAG (Fig. S2A).

The third example case is a mapping between the domain “SAM-dependent methyltransferase RsmB/NOP2-type” (IPR001678) and the molecular function term “C-methyltransferase activity” (GO:0008169). These two terms were mapped to each other with a co-occurrence similarity (S) value of 0.39, on a total of 24 proteins (n). The “methyltransferase activity” of the IPR001678 domain is verified by a manually curated InterPro2GO association with the GO term of the same name (GO:0008168). GO:0008168 is also related (i.e., with is_a relationship) to the Domain2GO mapped GO:0008169 as its direct parent. As the names and the parent-child relationship of the two terms suggest, “C-methyltransferase activity” defines a more specific function. The InterPro database description of the IPR001678 domain is as follows: “The C-terminal domain of ribosomal RNA cysteine methyltransferases is highly conserved in archaeal, bacterial, and eukaryotic proteins” (URL: https://www.ebi.ac.uk/interpro/entry/InterPro/IPR001678/). The proteins mentioned in this description could be associated with the “C-methyltransferase activity” function thanks to the IPR001678-GO:0008169 mapping in Domain2GO. Also, as shown in Fig. S2B, we were able to find links between IPR001678 and some other molecular function and biological process terms that are also closely related to the InterPro2GO-associated GO:0008168 term.

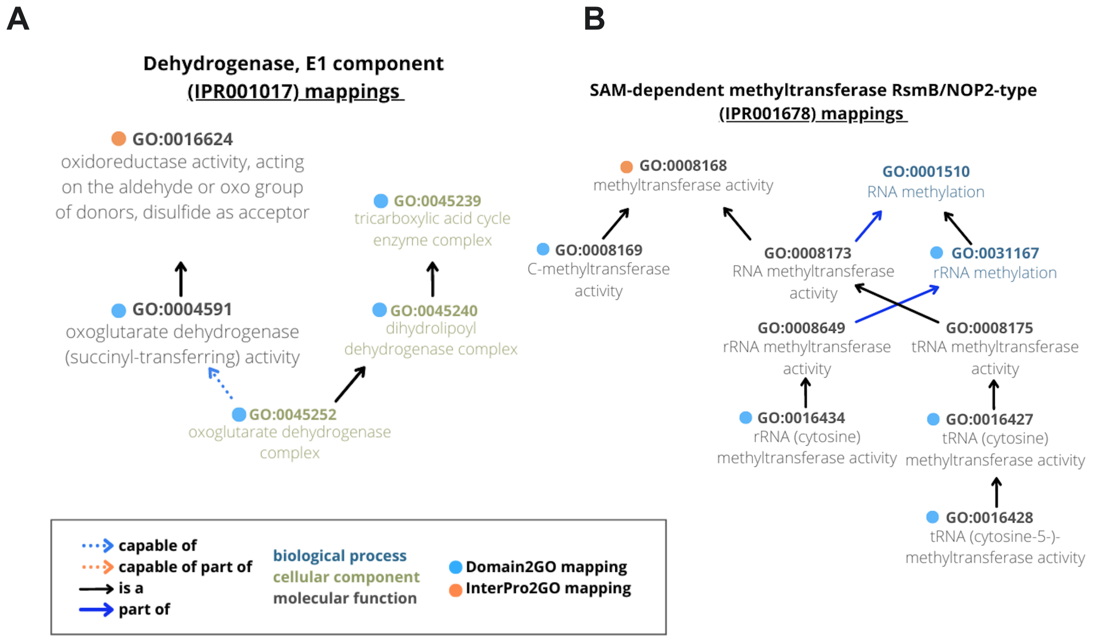

Figure S2. A case study comparing Domain2GO with curated InterPro2GO annotations on selected domains. (A) Case 2: GO terms associated with Dehydrogenase, E1 component (IPR001017) domain. **(B) Case 3:** GO terms associated with SAM-dependent methyltransferase RsmB/NOP2-type (IPR001678) domain.

6. Minimum semantic distance (Smin) results on the CAFA3 benchmark set

Table S4. Protein function prediction performance results (Smin) on the CAFA3 benchmark set for 3 ontologies in full evaluation mode. Lower minimum semantic distance (Smin) values indicate higher performance. The best scores for each ontology are shown in bold.

	Model	MFO		Model	BPO		Model	CCO	
		Smin	Coverage		Smin	Coverage		Smin	Coverage
**CAFA3 Top 10**	ZhuLab2	6.26	1.00	ZhuLab2	**14.78**	1.00	DessimozLab	**5.12**	0.88
	Orengo-FunFams-2	6.47	0.85	INGA-Tosatto	15.03	0.99	INGA-Tosatto	5.15	0.99
	Zhang-Freddolino-Lab	6.93	0.99	Orengo-FunFams-3	15.13	0.71	ZhuLab1	5.20	1.00
	INGA-Tosatto	6.93	1.00	Zhang-Freddolino-Lab	15.22	1.00	Argot25ToppoLab	5.21	1.00
	TurkuBioNLP1	6.95	0.79	Temple	15.32	0.99	kiharalab4	5.27	1.00
	tianlab1	7.11	0.92	Jones-UCL-CW	15.36	1.00	Holm1	5.27	0.93
	DeepMaster	7.26	1.00	cbrcborg	15.37	0.90	DeepMaster	5.28	1.00
	Holm1	7.30	0.88	DeepMaster	15.38	1.00	Orengo-FunFams-2	5.30	0.87
	Jones-UCL-CW	7.32	1.00	Ritchie-LORIA	15.46	0.88	Jones-UCL-CW	5.32	1.00
	goat	7.43	0.98	kiharalab4	15.48	1.00	cbrcborg	5.42	0.89
**Domain-based**	Domain2GO-S	6.22	0.58	Domain2GO-S	15.93	0.58	Domain2GO-S	5.86	0.52
	Domain2GO-E	7.12	0.53	Domain2GO-E	17.06	0.50	Domain2GO-E	6.20	0.42
	DomFun	**6.19**	0.41	Domfun	16.01	0.46	Domfun	5.57	0.49
	InterPro2GO	6.47	0.39	InterPro2GO	16.85	0.23	InterPro2GO	6.48	0.20
**Baseline**	Naive	8.85	0.93	Naive	16.20	0.97	Naive	5.86	0.97
	BLAST	8.09	0.93	BLAST	16.87	0.97	BLAST	5.98	0.97

7. Organism-specific comparative analysis of Domain2GO performance

The coverage values of all methods, representing the proportion of proteins for which each method provided predictions out of the total number of proteins in the benchmark set, are displayed within the bars in all figures.

In the CAFA3 paper, organism-specific performance comparisons for a total of nine different organisms were provided (Zhou et al. 2019). However, in our study, we focused on analyzing and presenting performance comparisons for the organisms: *Mus musculus, Rattus norvegicus, Drosophila melanogaster* and *Dictyostelium discoideum*. We selected these organisms based on Domain2GO's competitive performance levels. We also included *Homo sapiens* since it is the most commonly occurring organism in the benchmark set and also the target of nearly all biomedical applications. Notably, for the remaining four organisms -*Arabidopsis thaliana, Escherichia coli K12, Danio rerio, and Candida albicans (strain SC5314 / ATCC MYA-2876)*- Domain2GO generally performed relatively poor. In most cases, it achieved results comparable to baseline methods but fell short of the top-performing models in CAFA3. This difference in performance may be attributed to variations in the extent and quality of available functional annotations for protein domains in these organisms. Proteomes of organisms such as mouse, rat, fruit fly, and human benefit from more comprehensive and higher-quality annotation data, making it possible to train effective prediction models. Another possible factor contributing to Domain2GO's performance across these organisms may be the concept of domain modularity. Our analysis showed that the five organisms on which Domain2GO performed better (Figure S3-S7) had higher modularity, characterized by a median domain count of 2 per protein. In contrast, the other four organisms had a median domain count of only 1 per protein, which means that those domains are more likely to be eliminated during the thresholding operations in the domain-GO mapping formation. Domain2GO's scoring mechanism heavily relies on assessing the frequency of domain annotations across proteins, and when the modularity is low, Domain2GO may assign predictions with lower reliability or give no predictions at all.

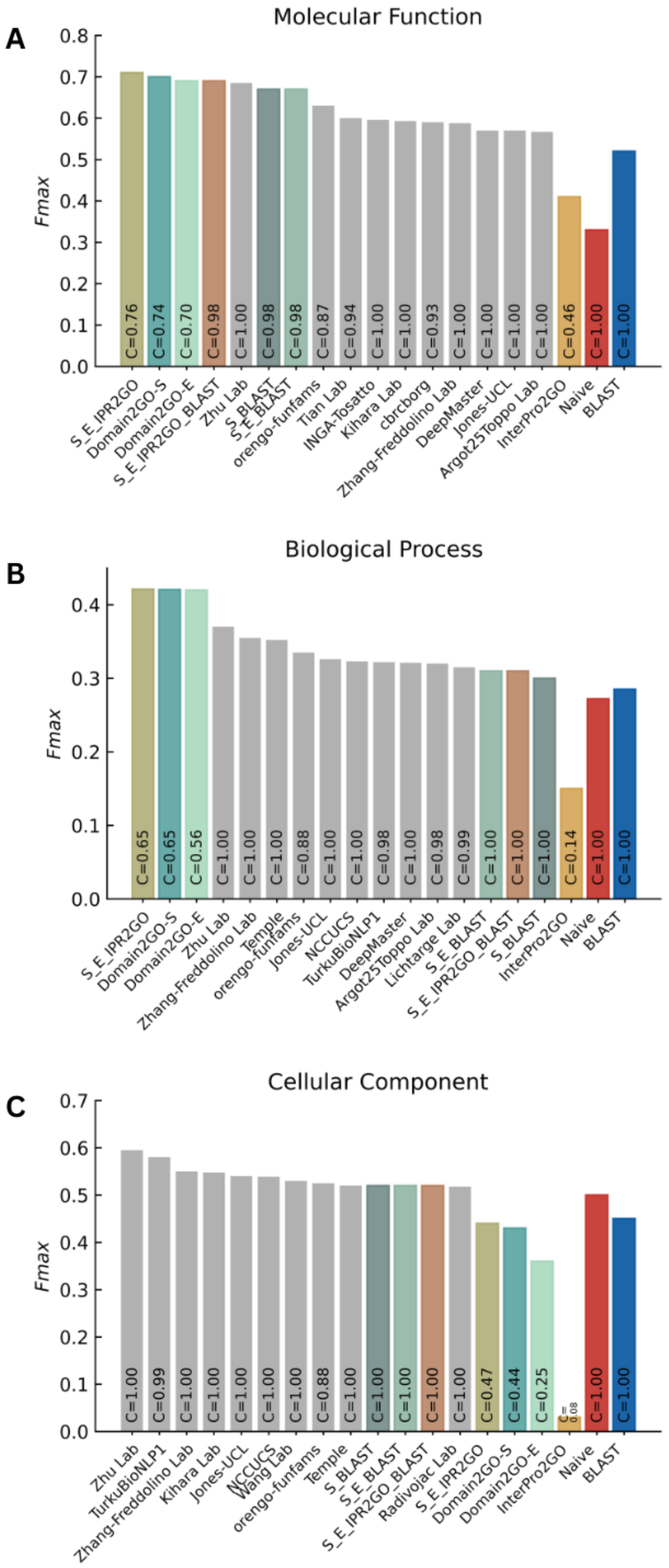

Figure S3. Protein function prediction performance results (Fmax) for *Mus musculus* on the CAFA3 benchmark set for 3 ontologies. (A) molecular function ontology, **(B)** biological process ontology, and **(C)** cellular component ontology.

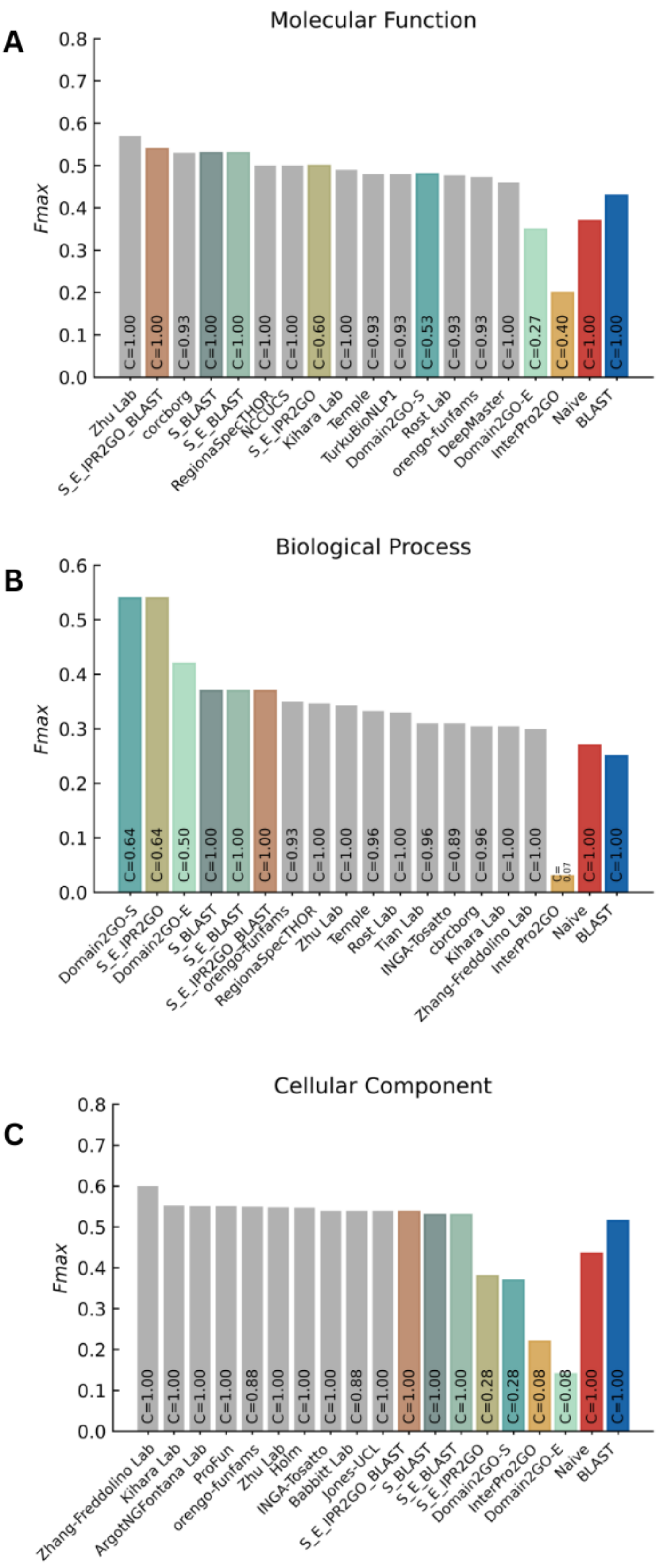

Figure S4. Protein function prediction performance results (Fmax) for *Rattus norvegicus* on the CAFA3 benchmark set for 3 ontologies. (A) molecular function ontology, **(B)** biological process ontology, and **(C)** cellular component ontology.

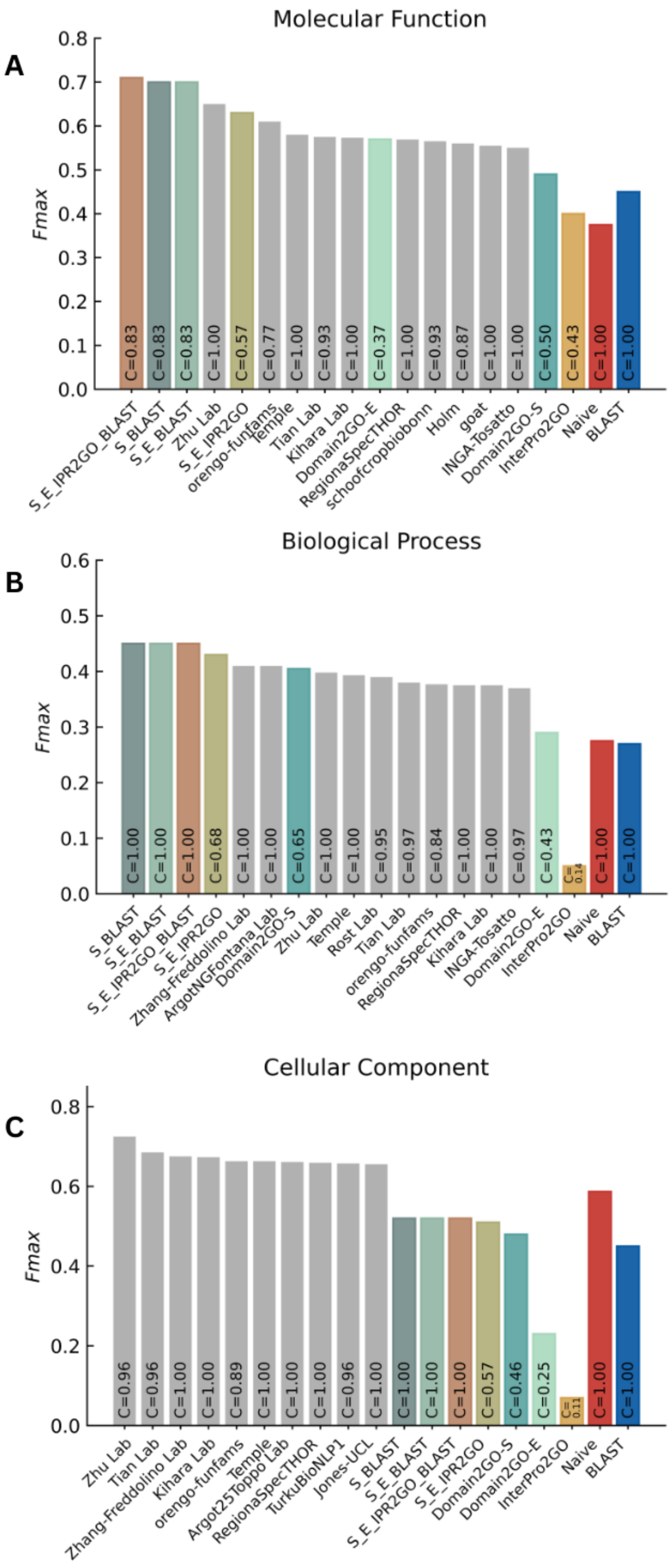

Figure S5. Protein function prediction performance results (Fmax) for *Drosophila melanogaster* on the CAFA3 benchmark set for 3 ontologies. (A) molecular function ontology, **(B)** biological process ontology, and **(C)** cellular component ontology.

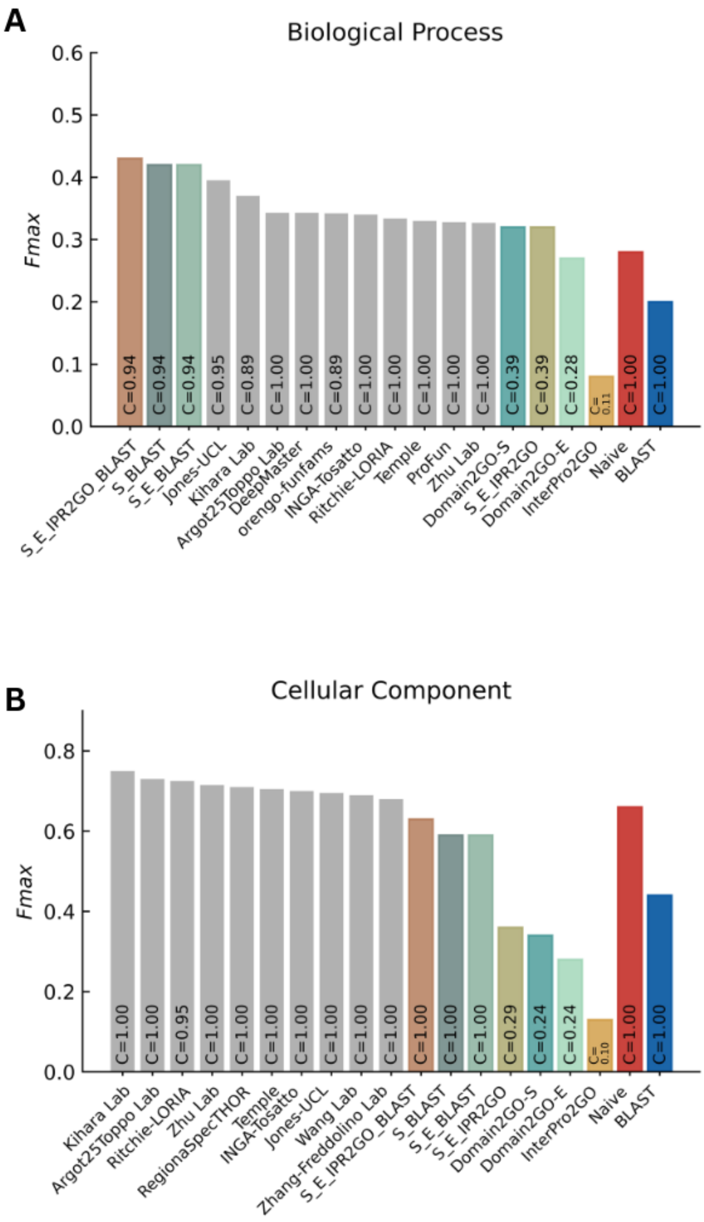

Figure S6. Protein function prediction performance results (Fmax) for *Dictyostelium discoideum* on the CAFA3 benchmark set for 2 ontologies. (A) biological process ontology, and **(B)** cellular component ontology. Molecular function ontology was not included in the evaluation due to the absence of molecular function term annotations for the proteins from this organism in the benchmark set.

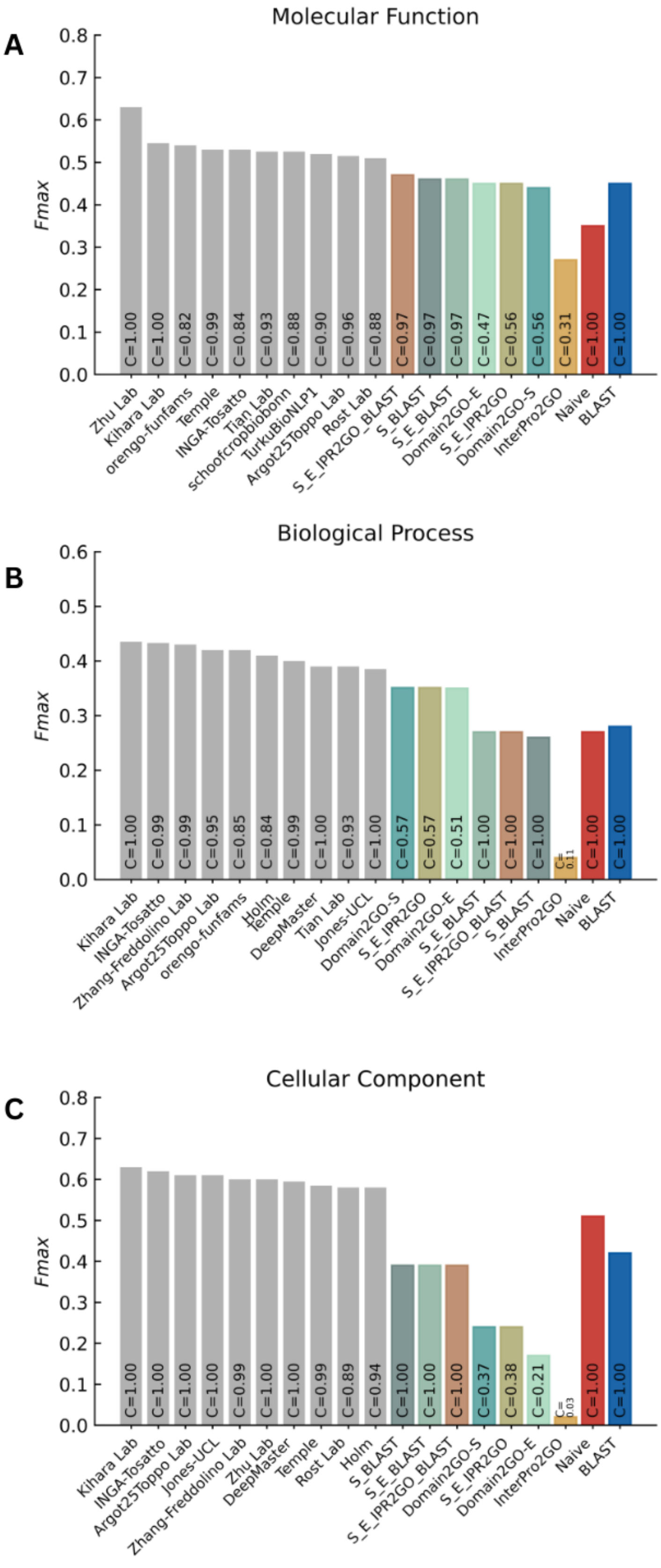

Figure S7. Protein function prediction performance results (Fmax) for *Homo sapiens* on the CAFA3 benchmark set for 3 ontologies. (A) molecular function ontology, **(B)** biological process ontology, and **(C)** cellular component ontology.

8. Correspondence between Domain2GO and BLAST predictions

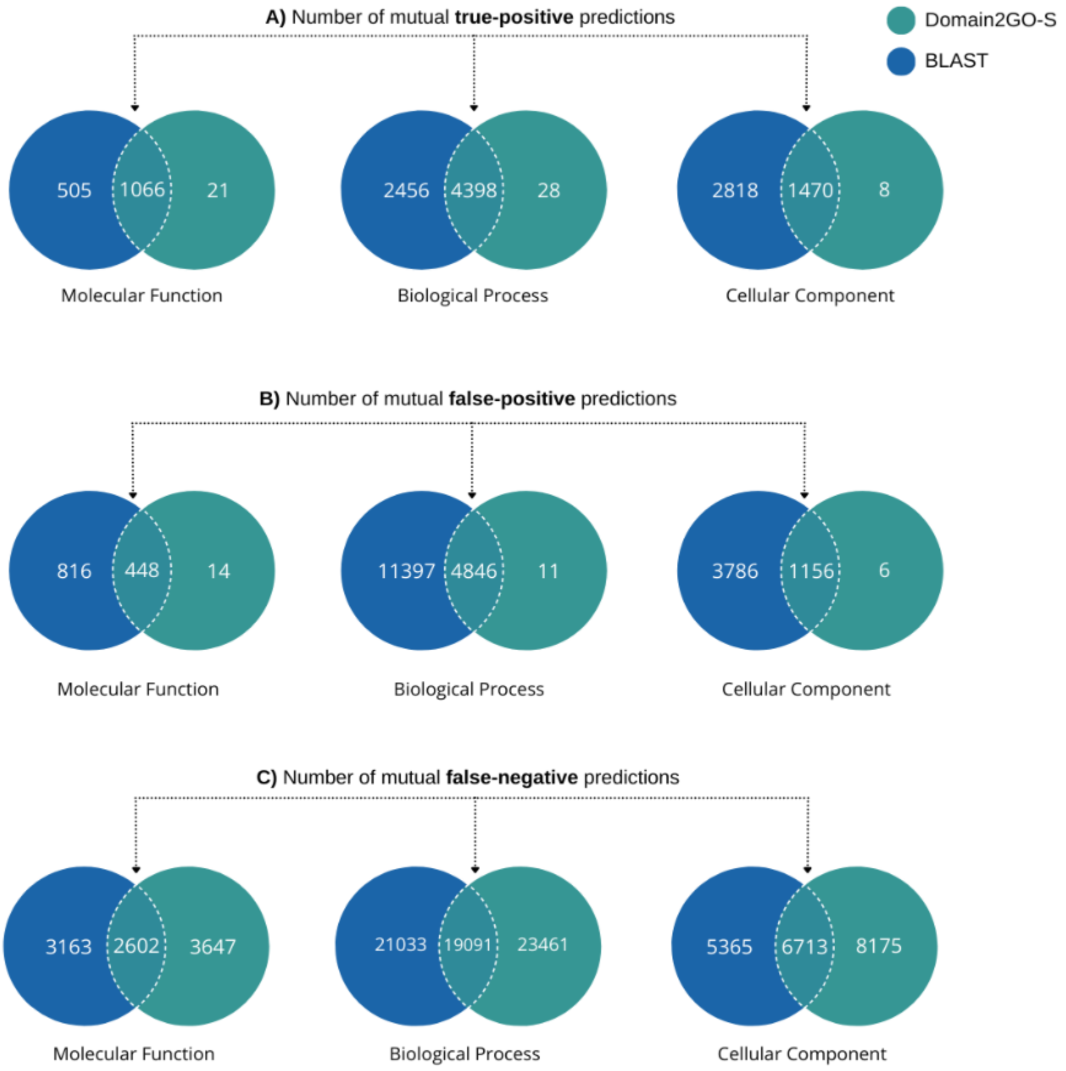

Figure S8. The analysis of correspondence between Domain2GO and BLAST predictions. The number of **(A)** true positives, **(B)** false positives, **(C)** false negatives of Domain2GO-S-only, mutual, and BLAST-only predictions at the optimal thresholds. The mutual positive predictions are the protein-GO term pairs, which are predicted as positive by either the Domain2GO-S or BLAST methods. The mutual negative predictions are the protein-GO term pairs, which are predicted as negative by both the Domain2GO-S and BLAST methods.

9. Performance comparison of Domain2GO with Best-Performing Model with User-Friendly Tool Availability

Table S5. Protein function prediction performance results (Fmax) of Domain2GO and the top-performing CAFA3 models with available tools in each ontology on the CAFA3 benchmark set. The best scores for each ontology and evaluation mode combination are shown in bold.

Model/Lab	GO category and evaluation mode					
	MFO		BPO		CCO	
	Partial	Full	Partial	Full	Partial	Full
Kihara Lab	-*	**0.53**	-	**0.38**	**0.61**	**0.61**
Holm	0.55	0.51	0.40	**0.38**	0.60	-
schoofcropbiobonn	0.55	0.52	-	-	-	-
Domain2GO-S	0.63	0.48	0.46	0.36	0.52	0.31
Domain2GO-E	**0.68**	0.46	**0.49**	0.35	0.59	0.22

* Scores denoted with "-" were not available due to the fact that the method of interest could not make it to the top 10 in CAFA3 for the respective evaluation mode - performance metric combination.

Supplementary References

Bunik VI, Degtyarev D. 2008. Structure-function relationships in the 2-oxo acid dehydrogenase family: substrate-specific signatures and functional predictions for the 2-oxoglutarate dehydrogenase-like proteins. Proteins. 71(2):874–890. doi:10.1002/prot.21766.

Tretter L, Adam-Vizi V. 2005. Alpha-ketoglutarate dehydrogenase: a target and generator of oxidative stress. Philos Trans R Soc Lond B Biol Sci. 360(1464):2335–2345. doi:10.1098/rstb.2005.1764.

Zhou N, Jiang Y, Bergquist TR, Lee AJ, Kacsoh BZ, Crocker AW, Lewis KA, Georghiou G, Nguyen HN, Hamid MN, et al. 2019. The CAFA challenge reports improved protein function prediction and new functional annotations for hundreds of genes through experimental screens. Genome Biol. 20(1):244. doi:10.1186/s13059-019-1835-8.
